# Targeted phagocytosis of TNF-α by CAR macrophages restores immune homeostasis in arthritis

**DOI:** 10.7150/thno.132172

**Published:** 2026-08-24

**Authors:** Mei-Qi Wang, Jie Zhu, Chun-Yu Liu, Xiao-Lin Yu, Ya-Ru Huang, Lei Zhang, Shi-Yu Liang, Lixia Zhang, Hao-Tian Wang, Hongbo Cheng, Jia-Qian Chai, Xi-Xiu Xie, Rui-Tian Liu

**Affiliations:** 1 State Key Laboratory of Biopharmaceutical Preparation and Delivery, Institute of Process Engineering, Chinese Academy of Sciences, Beijing 100190, China.; 2 University of Chinese Academy of Sciences, Beijing 100049, China.; 3 College of Veterinary Medicine, Shandong Agricultural University, Tai'an, Shandong, 271018, China.; 4 Rheumatology Department, the first affiliated hospital of Zhengzhou University, Zhengzhou, Henan, 450052, China.; 5 Department of Endocrinology, the First Affiliated Hospital of Zhengzhou University, Zhengzhou 450052, China.; 6 Department of Chemical and Biological Engineering, University of British Columbia, 2360 East Mall, Vancouver, BC V6T 1Z3, Canada.

**Keywords:** mer tyrosine kinase, rheumatoid arthritis, macrophages, TNF-α, inflammation resolution

## Abstract

**Rationale:**

Dysregulated phagocytic clearance within the synovial microenvironment contributes to persistent inflammation and impairs immune homeostasis in rheumatoid arthritis (RA). Because MerTK-positive macrophages are essential for efferocytosis and inflammation resolution, but are functionally impaired in RA, this study aimed to engineer macrophages capable of enhancing tumor necrosis factor-α (TNF-α) clearance and restoring inflammation-resolving macrophage function.

**Methods:**

We generated anti-TNFα chimeric antigen receptor macrophages (CAR-M) by replacing the MerTK antigen-binding domain with a TNF-R1 fragment. The phagocytic and degradative capacity of CAR-M toward soluble TNF-α was assessed, together with activation of Rho GTPases Rac1, Cdc42, and RhoA. Macrophage inflammatory mediator production, phenotypic polarization, SOCS1/3, NF-κB, and MAPK signaling were analyzed. The effects of CAR-M cells were further compared with adalimumab and control treatments under synovial fluid stimulation from patients with arthritis and in mice with collagen-induced arthritis (CIA).

**Results:**

Anti-TNFα CAR-M showed markedly enhanced phagocytosis and degradation of soluble TNF-α, accompanied by Rac1, Cdc42, and RhoA activation. CAR activation reduced inflammatory mediator production, including soluble TNF-α, interleukin-1 β (IL-1β), and interleukin-6 (IL-6), while increasing interleukin-10 (IL-10) secretion. This functional shift was associated with polarization toward an inflammation-resolving MerTK^+^CD206^+^ phenotype, SOCS1/3 upregulation, and NF-κB and MAPK pathway inhibition. Under stimulation with synovial fluid from arthritis patients, anti-TNFα CAR-M decreased TNF-α and IL-6 levels and drove macrophages toward an M2-like anti-inflammatory state. In CIA mice, local *in situ* CAR-M cell treatment produced stronger therapeutic effects than adalimumab or other controls, significantly alleviating inflammatory activation, clinical symptoms, and joint pathologies.

**Conclusions:**

Anti-TNFα CAR-M exert therapeutic effects via a dual mechanism: direct TNF-α clearance and synovial macrophage reprogramming toward a reparative phenotype. This synthetic biology strategy highlights the potential of precise cell engineering to correct phagocytic deficits and resolve chronic inflammation in RA.

## Introduction

Rheumatoid arthritis (RA), a systemic autoimmune disorder, affects approximately 1% of the global population 1. This disease involves sustained synovial inflammation and may affect extra-articular tissues, resulting in pain, joint damage, and disability 2. Although RA has been widely investigated, its precise pathogenic mechanism has not been fully defined. Increasing evidence has shown that macrophages are the major contributors to the development and progression 3. Activated synovial macrophages serve as a major cellular source of inflammatory mediators, among which tumor necrosis factor-α (TNF-α) acts as a pivotal pathogenic driver of RA 4. The elevated TNF-α within RA synovial tissue and circulation, as a primary driver of inflammation, disrupts the homeostatic equilibrium between inflammatory and resolving cytokine programs 5,6, and promotes additional inflammatory mediators, including interleukin-1β (IL-1β), chemokines, and proteases. Consequently, TNF-α clearance has become a clinically established strategy for managing RA. However, approximately one-third of patients initially fail to respond to such therapies 7. Nearly 50% of initial responders experience disease flares within one year of treatment cessation 8-10. Therefore, effective therapeutic strategies for treating RA are required. Arthritis progression is driven by complex interactions between genetic susceptibility, inflammatory signaling, cell death programs, and local tissue microenvironmental changes. Multiple chondrocyte death-related processes, including apoptosis, pyroptosis, ferroptosis, and dysregulated autophagy, contribute to osteoarthritis (OA) progression 11. Emerging therapeutic strategies have increasingly focused on remodeling the joint microenvironment. For example, MXene photothermally injectable hydrogel microspheres have been reported to mitigate OA by modulating the physicochemical inflammatory microenvironment 12, whereas low-intensity pulsed ultrasound has been explored as a non-invasive approach to regulate inflammation, cartilage metabolism, and tissue repair. These advances highlight the importance of developing microenvironment-oriented therapeutic strategies for arthritis 13. MerTK, a phagocytic receptor that mediates efferocytosis and suppresses inflammation through the upregulation of SOCS1/SOCS3 (key inhibitors of pro-inflammatory signaling), is central to inflammation resolution 14. MerTK (CD206) macrophage subpopulations constitute the dominant synovial subsets in healthy joints. During inflammatory arthritis, such as RA or experimental models, synovial macrophages acquire a MerTK^-^CD206^-^ state that produces high amounts of TNF-α, IL-1β and IL-6 and expands with disease severity 15. In addition, MerTK blockade increases the release of inflammatory cytokines and aggravates joint pathologies and symptoms 16. Furthermore, MerTK⁺CD206⁺ synovial macrophage enrichment predicts low relapse risk after biologic-free discontinuation in RA patients achieving remission with TNF-α inhibitors 3. Therefore, MerTK⁺ macrophage drives clinical remission in RA by forming an anti-inflammatory lining barrier and restoring synovial immune homeostasis. Therapeutic MerTK⁺ macrophage modulation may support RA therapy and help sustain remission 3.

Chimeric antigen receptor (CAR) engineering has become an important modality in cellular immunotherapy, particularly cancer treatment 17. Here, we developed a chimeric antigen receptor macrophage (CAR-M) approach designed to capture TNF-α and re-establish immune balance, testifying the therapeutic effect of CAR-M on collagen-induced arthritis (CIA) mice.

## Materials and Methods

### Animals

Male *DBA/1* mice aged 6–8 weeks were obtained from Beijing Huafukang Biotechnology (Beijing, China). All mice were housed in groups with ad libitum food and water access and maintained at 22 ± 2 °C and 45 ± 10% humidity under a reversed 12-h light/dark schedule. All animal experiments were performed according to the China Public Health Service Guide for the Care and Use of Laboratory Animals. The experiments involving mice and protocols were approved by the Institutional Animal Care and Use Committee of the Institute of Process Engineering.

### Collagen-induced arthritis (CIA) animal model

All the animal experimental procedures were approved by the Animal Ethics Committee of the Institutional Animal Care and Use Committee of the Institute of Process Engineering, Chinese Academy of Sciences (approval no. IPEAECA2025168). All experiments were performed in accordance with the institutional guidelines and relevant national regulations for the care and use of laboratory animals. CIA was induced according to a published protocol 18. Briefly, chicken type II collagen (Chondrex, Redmond, WA, USA) was dissolved and emulsified in an equal volume of complete Freund’s adjuvant (1 mg/mL; Chondrex). Emulsion (0.1 mL) was intradermally injection into the base of the mouse tail. Three weeks later, a booster injection containing a mixture of incomplete Freund’s adjuvant (Chondrex, Cat# 20012) and type II collagen emulsion (1 mg/mL) was administered intradermally into the mouse tail. RA progression was monitored every three days. For treatment grouping, the booster immunization was designated as day 0. Five days before the booster immunization (Day -5), CIA mice received a local intra-articular injection of AAV-CAR into the joint cavity and were designated as the CIA-CAR therapeutic group. Mice with CIA receiving an equivalent dose of AAV-nsCAR were used as disease control vectors (CIA-nsCAR). To evaluate the biosafety of AAV-CAR in non-arthritic animals, age-matched wild-type* DBA/1* mice were locally injected with the same dose of AAV-CAR and designated as the WT-CAR biosafety control group. In addition, beginning on day 7 after the booster immunization, adalimumab (Selleck Chemicals, Houston, TX, USA) was included as a pharmacological comparator based on its prior use in murine CIA 19.

### Assessment of paw swelling and arthritis score

In the CIA and WT mouse groups, the anti-TNFα CAR and nsCAR plasmid delivered by anti-CD11b AAV2 were locally administered by intra-articular injection into the ankle joint of the mice at a dose of 1 × 10⁹ viral genomes (VG) on Day -5. Phosphate-buffered saline (PBS) was used as the control. Hind paw thickness was measured using digital calipers. The mice were scored every 3 days using the following scale: 0, normal; 1, mild swelling and redness confined to the tarsals or ankle joint; 2, mild swelling and redness extending from the ankle to the tarsals; 3, moderate swelling and redness extending from the ankle to the metatarsal joints; and 4, severe erythema and swelling involving the ankle, foot, and digits. Individual paw scores were summed for each CIA mouse paw.

### Cell culture

Bone marrow-derived macrophages (BMDMs) were cultured in Roswell Parks Memorial Institute (RPMI) 1640 (Gibco, USA, CA) containing 10% FBS, 1% penicillin–streptomycin, and 20 ng/mL recombinant mouse M-CSF (20 ng/mL) under standard conditions (37 ℃, 5% CO_2_, 95% humidity) for 7–10 days prior to experiment use. HEK293T cell lines were purchased from ATCC (ATCC Cat# CRL-3216, RRID: CVCL_0063) cultured in high-glucose DMEM containing 10% FBS and antibiotics under 37 °C and 5% CO_2_ conditions. HEK293T cells and BMDMs were confirmed to be free of mycoplasma using a Myco-Lumi luminescence kit (Beyotime, Shanghai, China, C0297S) (Figure S1).

### Preparation of lipopolysaccharide (LPS)-conditioned medium (CM)

BMDMs were differentiated and stimulated with LPS using a previously described procedure 20. Briefly, cells were cultured in the presence of M-CSF (20 ng/mL) for 7–10 days and the medium was replaced with fresh medium without M-CSF. The cells were cultured for 24 h and the macrophages were exposed to 1 μg/mL LPS for another 12 h. Conditioned medium was collected, centrifuged, and stored in aliquots at -80 ℃.

### Plasmid construction

The plasmids used in this study were generated by custom cloning at Sangon Biotech Co., Ltd. (Shanghai, China). The following two expression constructs were used:

(1) CD68-TNFR1^30-212^-Myc-^276-994^MerTK-HA-BGH polyA

In this construct (named anti-TNFα CAR), enhanced CMV promoter sequence in MerTK expression plasmid (purchased from Sino Biological Inc., #MG50514-ACG) was replaced with CD68 promoter (macrophage-specific promoter) sequence synthesized from Sangon. A signal peptide was introduced upstream for direct membrane localization. The ligand-binding domain gene corresponding to amino acids (AAs) 19-275 of MerTK was replaced with a TNFR1^30-212^ gene sequence. An HA tag was added to enable receptor detection along with–^276-994^MerTK before the stop codon and bovine growth hormone (BGH) poly-A signal.

(2) CD68-ns-scFv-^276-994^MerTK-HA-BGH polyA

In this construction (named nsCAR), TNFR1^30-212^ fragment gene sequence in plasmid (1) was exchanged for a non-specific scFv-encoding sequence to maintain identical molecular weights relative to the anti-TNFα CAR.

### Construction of macrophage-targeted adeno-associated virus-2 (AAV2) capsid protein plasmids and AAV purification

AAV capsid proteins VP1, VP2, and VP3, arise from alternative start codons within the open reading frame (ORF). To reduce non-specific infectivity, arginine at residues 585 and 588 was mutated to alanine to decrease its binding ability to heparan sulfate proteoglycans (HSPG).

The AAV2 targeting CD11b was engineered using previously established methodologies 21. Briefly, the VP1 GH2/GH3 loop forms an exposed region on the AAV2 capsid. Through genetic modification, the seven AAs in the GH2/GH3 loop of AAV2 (AA 453–459) were replaced with a flexible peptide-linked anti-CD11b nanobody consisting of 148 AA residues for targeting macrophages, and this was named pAAV2- antiCD11b -VP1. Because the expression of VP2 and VP3 would be drastically reduced after inserting the large fragment, the start codon ATG was mutated to AAG so that the RC2 plasmid could only express VP2 and VP3 and was named pAAV2-R585/588A-VP23. The recombinant AAV targeting CD11b was named anti-CD11b-AAV2. For anti-CD11b-AAV2 production, four plasmids, namely pAAV2- anti CD11b-VP1, pAAV2-R585/588A-VP23, pAAV-helper, and the shuttle plasmid carrying the target gene, were co-transfected into 293T cells. The modification and packaging of the target genes were completed in 293T cells. Finally, AAV was obtained by iodixanol density gradient centrifugation, followed by ultrafiltration and buffer exchange. Virus purification and preparation were performed at Obio Technology. To investigate the effect of MOI on the transfection efficiency of the CAR in BMDMs, cells were transduced with varying MOIs of AAV. Following 72 h of expression, BMDMs were stimulated using 20 ng/mL of TNF-α and incubation continued. The phagocytic and digestive capacity of TNF-α by anti-TNFα CAR-M were then detected within a 12 h window.

### Endogenous TNFR1/TNFα blockade assay

To distinguish CAR-mediated uptake from endogenous TNFR1-dependent effects, BMDMs were treated with a function-blocking anti-TNFR1 antibody under conditions validated in the present study 22. BMDMs were pretreated with an anti-TNFR1 blocking antibody (eBioscience™, Thermo Fisher Scientific, Cat# 16-1202-81) at 5 μg/mL for 30 min at 37 °C. After pretreatment, the cells were washed to remove unbound antibodies and then transfected with CAR or nsCAR plasmids, according to the protocol described above. After 72 h of transfection, cells were incubated with Cy5-labeled TNF-α at 20 ng/mL for 30 min. Cy5-TNF-α association with CAR-M or nsCAR-M was then evaluated by confocal fluorescence imaging. The assay was designed to reduce endogenous TNFR1-mediated TNF-α association and to compare CAR-dependent TNF-α binding under the same TNFR1-blocked background. For endogenous TNF-α neutralization, CAR- or nsCAR-transfected BMDMs were pretreated with a neutralizing anti-TNFα antibody at 15 μg/mL for 1 h at 37 °C before incubation with Cy5-labeled TNF-α. The neutralization experiment was designed and validated in the present study; however, it should not be adapted from 23. After pretreatment, unbound antibody was removed by washing before Cy5-TNF-α incubation at 20 ng/mL for 30, 60, 240, 300 min. Cy5-TNF-α association and degradation were evaluated by confocal fluorescence imaging based on Cy5 fluorescence intensity.

### Flow cytometry

The effects of CAR-M on CAR expression and exogenous TNF-α (labeled with Cy5) phagocytosis was assessed by flow cytometry with sequential staining 17. Briefly, after fixation for 20 min with 4% PFA, BMDMs were treated with 0.3% Triton X-100 and labeled with an anti-Cy5 primary antibody (Abcam, Cat# ab52061, RRID: AB_869295) and a PE-conjugated anti-mouse secondary antibody (Biolegend, #406421, RRID: AB_2563484). Using identical staining and detection methodology, the following parameters were quantified: The positive anti-TNFα CAR (or nsCAR) expression rate in BMDMs, and polarization status of BMDMs induced by CAR under inflammatory conditions. The following antibodies were used: APC-CD11b (Biolegend Cat# 101211, RRID: AB_312794), FITC-F4/80 (BioLegend Cat# 123107, RRID: AB_893500), PE-CD86 (BioLegend Cat# 696805, RRID: AB_2876745), FITC-CD206 (BioLegend Cat# 162507, RRID: AB_3097515), HA (Abcam Cat# ab9110, RRID: AB_307019), and 488-Donkey-Mouse (Thermo Fisher Scientific Cat# A32766, RRID: AB_2762823). Data acquisition was performed using a Cyto FLEX LX (Beckman) and analysis was performed using FlowJo.

### Immunocytochemistry (ICC)

Glass slide cultures were rinsed three times using PBS, and the cells were fixed, permeabilized, and blocked using 4% PFA, 0.3% Triton X-100, and 10% normal donkey serum (NDS), respectively in PBS for 1 h at room temperature. Primary antibodies were applied overnight at 4 °C, followed by fluorophore-conjugated secondary antibodies (-488, -594 or -647) for 45 min and counterstained with Hoechst (Amsterdam) (Cell Signaling Technology, Danvers, MA, United States of America Cat# 4082, RRID: AB_10626776, 1:10,000) for 15 min at room temperature in dark, and then mounted on coverslips with anti-fade mounting medium. The following primary antibodies were used in this study: APC-CD11b (Biolegend Cat# 101211, RRID:AB_312794, 1:00), PE-CD86 (BioLegend Cat# 696805, RRID:AB_2876745, 1:100), FITC-CD206 (BioLegend Cat# 162507, RRID:AB_3097515,1:100), HA (Abcam Cat# ab9110, RRID:AB_307019,1:100); Myc (Santa Cruz, CA, United States of America Cat# sc-40, RRID:AB_627268, 1:100), LAMP1 (Abcam Cat# ab24170, RRID:AB_775978, 1:100), and TNF-α (Affinity Biosciences Cat# AF7014, RRID:AB_2835319, 1:100).

### Lysosomal inhibition assay

To determine whether internalized TNF-α was degraded through a lysosome-dependent pathway, a chloroquine (CQ)-based lysosomal inhibition assay was performed using CQ to inhibit lysosomal degradation of internalized TNF-α or macrophage-internalized substrates. Briefly, CAR-M or nsCAR-M were incubated with recombinant TNF-α under the indicated conditions to allow TNF-α binding and uptake. For lysosomal inhibition, cells were treated with CQ at 20 μM during the degradation phase. At the indicated time points, the supernatants were harvested and clarified by centrifugation to remove cell debris. The supernatant TNF-α was measured with a TNF-α enzyme-linked immunosorbent assay (ELISA) kit following the supplied protocol.

### Western blotting analysis

Cell lysates were separated using sodium dodecyl sulfate-polyacrylamide gel electrophoresis (SDS–PAGE) for western blotting and transferred onto nitrocellulose membranes. Membranes were blocked for 1 h at room temperature in 5% non-fat milk, the membrane was incubated with primary antibodies overnight at 4 °C. After washing with PBS containing 0.1% Tween-20 (PBST), horseradish peroxidase (HRP)-conjugated secondary antibodies were used at a concentration of 1:5,000 at room temperature. The bands in the immunoblots were detected by ECL on an Amersham (Chicago, United States) Imager 680 system (GE Healthcare, Little Chalfont, UK) and quantified using ImageJ software (RRID: SCR_003070). Membrane and cytosolic proteins were separated using the Membrane and Cytosol Protein Extraction Kit (Beyotime; #P0033). Primary antibodies to the following proteins were used: HA (Abcam, ab9110, 1:1000), Myc (Santa Cruz, #sc-40, 1:1000), MerTK (Abcam, #ab95925, RRID:AB_10863559, 1:1000), RhoA (Invitrogen, CA, USA, #PA5-87403, RRID: AB_2804123, 1:500), Rac1 (Invitrogen, #PA1-091X, RRID: AB_2539857, 1:500), Cdc42 (Invitrogen, #PA1-092, RRID: AB_2539858, 1:500), p-MerTK (Bioss, #bs18791R, 1:1000), p-STAT3 (Affinity Biosciences Cat# AF3293, RRID:AB_2810278), STAT3 (Affinity Biosciences Cat# AF6294, RRID:AB_2835144), p-JAK (Thermo Fisher Scientific Cat# MA5-36891, RRID:AB_2896826), JAK (Antibodies-Online Cat# ABIN362145, RRID: AB_10955670), SOCS1 (Sangon, #D260748, 1:1000), SOCS3 (Sangon, #D121242, 1:1000), p-IκB-α (Cell Signaling Technology, #2859T,1:1000), IκB-α (Beyotime, #AI096,1:1000), p-p65 (Abcam Cat# ab32536, RRID:AB_776751), p65 (Cell Signaling Technology, Cat# 8242, RRID: AB_10859369, 1:1000), p-PI3K (Affinity Biosciences Cat# AF3241, RRID: AB_2834667), PI3K (Affinity Biosciences Cat# AF6241, RRID: AB_2835340) AKT (Affinity Biosciences Cat# AF0791, RRID: AB_2834115), p-P38 (Affinity Biosciences Cat# AF4001, RRID: AB_2835330), P38 (Affinity Biosciences Cat# AF6456, RRID: AB_2835277), p-ERK1/2 (Affinity Biosciences Cat# AF1015, RRID: AB_2834432), ERK1/2 (Affinity Biosciences Cat# AF0155, RRID: AB_2833336), and β-actin (ZSGB-BIO, TA-09, 1:1000).

### Pull-down assay

GTP-bound RhoA and Cdc42/Rac1 were isolated using GST-Rhotekin-RBD and GST-PAK-PBD beads as previously described 24. Briefly, RBD and PBD fusion proteins were expressed in BL21(DE3) bacteria and captured on Glutathione Sepharose 4B (Cytiva, #17075601), protein lysates of CAR-M, nsCAR-M and BMDM treated with or without 20 ng/mL TNF-α for 24 h were mixed with 20 μL of PBD or RBD beads and incubated for 45 min at 4 °C. The beads were then washed, and the proteins bound to the beads were subjected to western blotting.

### ELISA

The levels of pro-inflammatory cytokines (IL-1β, TNF-α, and IL-6) and anti-inflammatory cytokines (IL-10 and IL-4) in ankle joint lysates from CIA mice and culture supernatants were measured with the indicated ELISA kits (Solarbio, Cat#SEKM-0034, SEKM-0007, SEKM-0002, SEKM-0010, SEKM-0005) following the manufacturer’s protocol. The absorbance was measured at 450 nm using a SpectraMax M5 microplate reader.

### mRNA sequencing

Total RNA was isolated using the TRIzol reagent (Invitrogen) according to the manufacturer’s protocol. The RNA concentration and purity were determined using a NanoDrop 2000 spectrophotometer (Thermo Scientific, CA, USA). RNA integrity was assessed using the Agilent (Santa Clara, CA, USA) 2100 Bioanalyzer (Agilent Technologies). Libraries were constructed using the VAHTS Universal V10 RNA-seq Library Prep Kit (Premixed Version), according to the manufacturer’s instructions. RNA-Seq library preparation and data analysis were performed by OE Biotech Co., Ltd. (Shanghai, China).

### Preparation of synovial fluid-conditioned medium (SF-CM)

Synovial fluid was obtained from patients with arthritis under aseptic conditions and immediately transported on ice. Sequential centrifugation at 2,000 g for 15 min was used at 4 °C to remove cells and debris. The supernatants were collected, diluted with complete culture medium when necessary, filtered through a 0.22 μm sterile filter, aliquoted, and stored at -80 °C until use. Before treatment, the synovial fluid samples were thawed on ice and mixed with complete macrophage culture medium at the indicated concentrations to generate a synovial fluid-conditioned medium. After obtaining informed consent, SF samples were obtained from patients with arthritis at the First Affiliated Hospital of Zhengzhou University. This study was approved by the Ethics Committee of the First Affiliated Hospital of Zhengzhou University (Approval No. 2026-KY-1196-001).

### RNA extraction and quantitative reverse transcription polymerase chain reaction (RT-qPCR)

Total RNA was extracted from ankle joints and cell lysates using TRIzol reagent. Reverse transcription was performed using the EasyQuick RT MasterMix (Cwbio, #CW 2019M, Taizhou, China) according to the manufacturer’s instructions. The relative gene expression of cDNA was detected by real-time qPCR using a 7500 Fast Real-time PCR system (Applied Biosystems, Foster City, CA, USA) and SYBR Selected Master Mix (Applied Biosystems, #4472908). β-actin served as the normalization control. The primers used were as follows:

TNF-α, 5′-CCCTCACACTCAGATCATCTTCT-3′

and anti-sense, 5′-GCTACGACGTGGGCTACAG-3′;

IL-6, 5′-TAGTCCTTCCTACCCCAATTTCC-3′

and anti-sense, 5′-TTGGTCCTTAGCCACTCCTTC-3′;

IL-1β, 5′-GCAACTGTTCCTGAACTCAACT-3′

and anti-sense, 5′-ATCTTTTGGGGTCCGTCAACT-3′;

IL-10, 5′-GCTCTTACTGACTGGCATGAG-3′

and anti-sense, 5′-CGCAGCTCTAGGAGCATGTG-3′;

IL-4, 5′-GGTCTCAACCCCCAGCTAGT-3′

and anti-sense, 5′-GCCGATGATCTCTCTCAAGTGAT-3′.

β-actin, 5′-TGTGATGGTGGGAATGGGTCAG-3′

and anti-sense, 5′-TTTGATGTCACGCACGATTTCC-3′

### Immunohistochemistry (IHC)

On day 25, the dissected ankles were fixed in 4% paraformaldehyde for 48 h, decalcified for 30 days in 15% neutral EDTA (Bomei Biotechnology Company, China), and embedded in paraffin.

For IHC analysis, 5 µm deparaffinized sections underwent citrate-buffer antigen retrieval (0.01 M, pH 6.0, 0.05% Tween-20) at 95 ℃ for 20 min. Sections were permeabilized and blocked with 10% NDS in 0.3% Triton-X 100 for 1 h at room temperature. Sections were incubated with PE-CD86, FITC-CD206, anti-HA antibodies at 4 ℃ overnight, followed by corresponding secondary antibodies conjugated to Alexa Fluor 488, -594 or -647, respectively. Sections were imaged using a Leica (Wetzlar, Germany) TCS SP8 confocal microscope. Images were processed and quantified using ImageJ software (National Institutes of Health, Bethesda, MD, USA, Version 1.52a). Instead of a single-cell analysis, the global fluorescence intensity within each field of view (FOV) was assessed. The images were first converted into 8-bit format. To separate the specific positive fluorescent signals from background noise, a consistent global thresholding strategy was applied across all images belonging to the same experimental batch. The threshold limit was determined using the *Default* auto-thresholding method in the ImageJ software. The Integrated Density (IntDen) and area fraction of specific fluorescence signals above the threshold were measured. A minimum of *3* representative images per group were analyzed in a blinded manner.

### Micro-computed tomography (Micro-CT)

The hind limbs from each group were scanned by micro-CT (Revvity, Quantum FXNEMO). Briefly, the samples were placed in the holder and scanned under the following conditions: scan resolution 7.5 μm, supply voltage 70 kV, current 0.08 mA, the scan progress was done by the acquisition software Cruiser and analyzed by analysis software Analyze14.0. The scanned data were reconstructed to generate 3D renderings of the ankle joints. For the segmentation of the 3D-volumes of the tibia, an evaluation script with adjusted grayscale thresholds of Caliper Micro-CT analysis tools was used. Bone mineral density (BMD), bone volume fraction (BV/TV), trabecular number (Tb.N), and trabecular separation (Tb.Sp) were analyzed using Avatar (version 1.6.5.3) and CTan analysis software.

### Hematoxylin and eosin (H&E) staining

For histopathological evaluation of the arthritic joints and major organs, tissues were collected after the mice were euthanized at the indicated experimental endpoint. Hind paw joints were dissected for bone and joint histology, and major organs, including the heart, liver, spleen, lungs, and kidneys, were harvested for systemic toxicity assessment.

For the joint and bone samples 25, excess soft tissue was carefully removed while preserving the ankle and tarsal joint structures. The specimens were fixed for 24–48 h in 4% PFA at room temperature. Because mineralized tissues require decalcification before paraffin sectioning, fixed joint samples were decalcified with gentle agitation in 10% EDTA at 4 °C. The decalcification solution was replaced every 2–3 days until the samples became sufficiently pliable for sectioning. EDTA-based decalcification was selected to preserve tissue morphology and staining quality, as recommended for rodent joint and bone histology in inflammatory arthritis studies. After decalcification, the samples were washed extensively in running water to remove residual EDTA. The tissues were processed using graded ethanol and xylene before paraffin embedding. Serial sections with a thickness of 5 μm were prepared using a rotary microtome. The sections of the ankle joints were oriented to include the ankle joint region and periarticular bone. The sections were placed on glass slides and dried.

Freshly isolated heart, liver, spleen, lung, and kidney tissues were fixed in 4% paraformaldehyde for 24–48 h. After routine dehydration, clearing, and paraffin embedding, tissue sections of 5 μm thickness were prepared. Paraffin sections were deparaffinized with xylene and rehydrated by decreasing the ethanol concentration in distilled water. Hematoxylin was used for nuclear staining, and the cells were rinsed and differentiated as appropriate. After washing, the sections were counterstained with eosin to visualize the cytoplasm and extracellular matrix. Stained sections were dehydrated using graded ethanol, cleared in xylene, and mounted with a neutral resin. Images were acquired using a bright-field microscope. Histology was assessed by a blinded evaluation. Inflammatory infiltration, synovial thickening, cartilage injury, and bone erosion were evaluated in joint sections. For the major organs, tissue morphology and signs of organ toxicity were examined.

### Tartrate-resistant acid phosphatase (TRAP) staining of bone sections

TRAP staining was performed on decalcified paraffin-embedded ankle joint sections to evaluate osteoclast formation and bone resorption in the arthritic joints. Briefly, hind paw or ankle joint tissues were fixed in 4% paraformaldehyde for 24–48 h, followed by decalcification in 10% EDTA solution (pH 7.2–7.4) with gentle agitation. After complete decalcification, samples were washed thoroughly, dehydrated using graded ethanol, cleared in xylene, and embedded in paraffin. Sections of 5 μm thickness were prepared and mounted on glass slides. The sections were then incubated with a TRAP staining solution prepared according to the manufacturer’s instructions. The staining reaction was performed at 37 °C for the recommended time until red-to-purple-red TRAP-positive cells were visible. After rinsing with distilled water, the sections were counterstained with hematoxylin or methyl green, dehydrated, cleared, and mounted. TRAP-positive osteoclasts were defined as multinucleated TRAP-positive cells located on or near the bone surface.

### Quantification of data from western blot experiments

Band density was measured using ImageJ software for western blot quantification. Only unsaturated images within the linear detection range were used for quantification. The local backgrounds were subtracted from each band. Target-band intensity was normalized against the matched loading control, such as β-actin, or β-tubulin, from the same lane. Phosphorylated proteins were normalized to the total protein content. The normalized values were then presented as a fold change relative to the control, set to 1. Quantification was performed in at least three independent experiments.

### Quantification of ICC

ICC images were analyzed using ImageJ software. Individual cells were delineated as region of interests (ROIs) based on DAPI staining or bright-field morphology. Mean fluorescence intensity and integrated density were measured for each cellular ROI. The background was calculated from nearby cell-free regions within the same field and subtracted from the raw ROI signal. At least 50–100 cells per condition were analyzed in three independent experiments in a blinded manner. The cell-level values were averaged for each independent experiment before statistical analysis.

### Quantification of IHC

Fluorescence quantification was performed at the field of view (FOV) level using ImageJ software. The images were split into individual channels and converted into 8-bit format. A consistent threshold was applied to all images within the same experimental batch to identify marker-positive signals. The integrated density and positive area fraction were measured for each FOV. Where appropriate, the fluorescence intensity was normalized to the number of DAPI-positive cells or total cell-covered area. Values from multiple fields were averaged for each independent experiment before statistical analysis.

### Statistical analysis

Data were analyzed using GraphPad Prism v.8.0.1 (RRID:SCR_002798). The statistical tests are specified in the figure legends. All data are represented as mean ± S.E.M. unless otherwise indicated. If the data were normally distributed, a one-way or two-way analysis of variance (ANOVA) was used with Tukey’s test for pairwise comparisons. In all cases, statistical differences were considered significant at * P < 0.05, ** P < 0.01, and *** P < 0.001, ****P < 0.0001.

## Results

### Expression of anti-TNFα CAR on BMDMs

**Figure 1:**
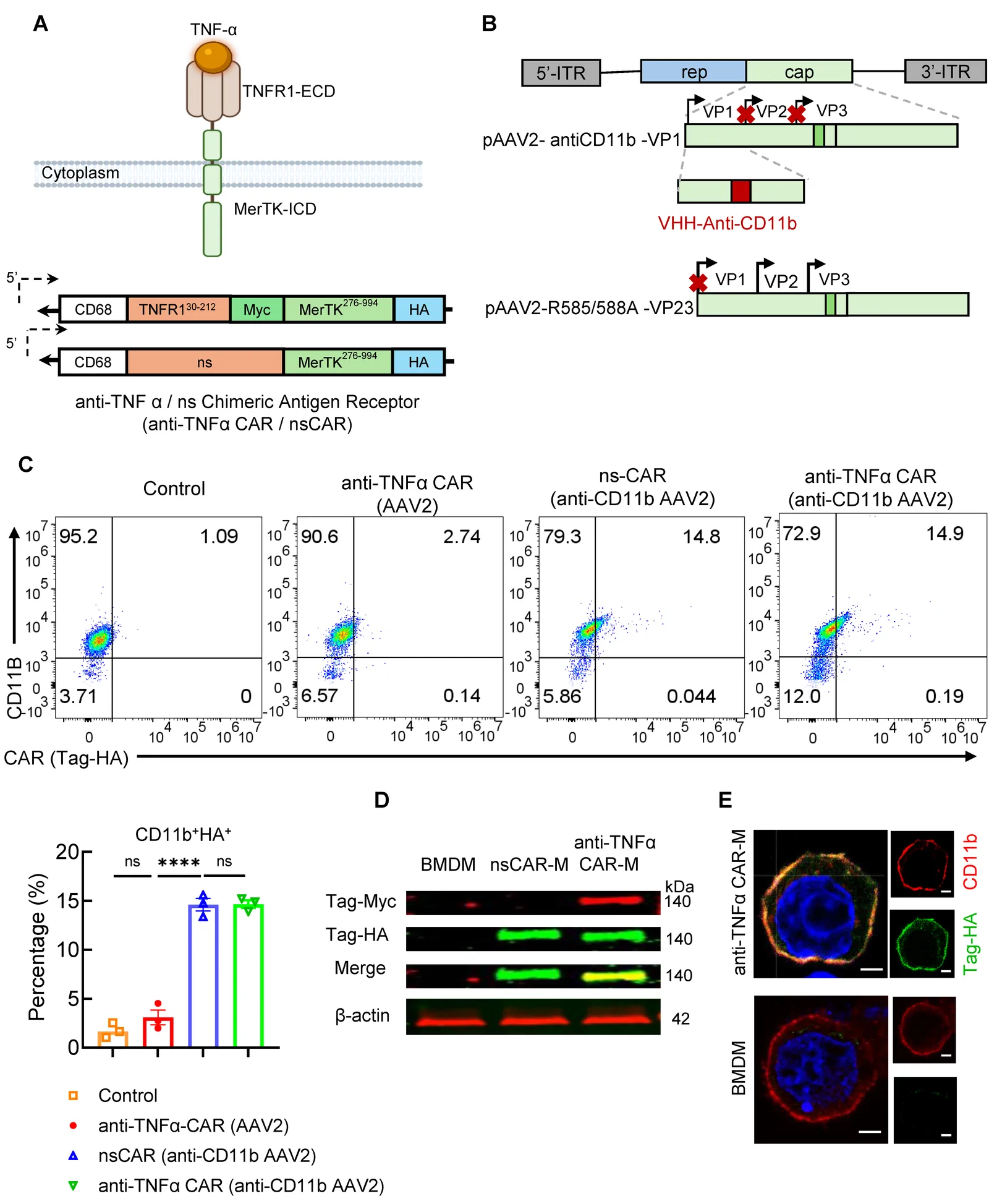
** The design of Anti-TNFα CAR and its expression on BMDMs. (A)** Schematic diagram of the anti-TNFα CAR construct. The expression is driven by the macrophage-specific CD68 promoter. The chimeric antigen receptor consists of the TNFR1 extracellular domain, alongside the MerTK transmembrane and intracellular signaling regions. To facilitate detection of TNFR1 and MerTK, a Myc tag and an HA tag were placed in the C-terminus of TNFR1 extracellular domain and MerTK domain, respectively. ECD: extracellular domain. ICD: intracellular domain. **(B)** Schematic diagram of AAV packaging plasmids engineered for targeting macrophages. To facilitate AAV2 targeting macrophages, the VP1 capsid protein was modified with CD11b nanobody. AAV packaging plasmid 1 (named pAAV2-antiCD11b-VP1) was constructed by inserting the CD11b nanobody sequence into the VP1 gene vector, with the VP2 and VP3 genes deleted. AAV packaging plasmid 2 (named pAAV2-R585/588A-VP23) was generated by deleting the VP1 gene while retaining the VP2 and VP3 genes. **(C)** Flow cytometry-based assessment of transduction efficiency of anti-TNFα CAR in BMDMs delivered by anti-CD11b-AAV2. AAV2 was served as a control. MOI = 150. n = 3. Data are shown as mean ± S.E.M, and statistical analysis used one-way ANOVA with Tukey’s post hoc test. **(D)** Fluorescence Western blot analysis of anti-TNFα CAR and nsCAR expression on BMDMs. Anti-HA antibody and anti-Myc antibody were used to detect MerTK and TNFR1, respectively. **(E)** Representative immunofluorescence images of anti-TNFα CAR-M and BMDMs stained for CD11b (red) and HA-tag (green). Scale bar represents 5 μm.

Macrophages express two distinct surface receptors for TNF-α, TNF-R1 and TNF-R2. TNF-R1 recognizes both soluble TNF-α and its membrane-associated form, whereas TNF-R2 preferentially responds to transmembrane TNF-α 22. Therefore, we chose TNF-R1 to construct CAR to bind to TNF-α. A ^30-212^TNFR1 exocellular domain or a nonspecific receptor control was fused to the intracellular MerTK^276-994^ signaling domains as anti-TNFα CAR or nsCAR. To determine whether the MerTK pathway activation was specifically induced by TNFR1 extracellular domain engagement, we generated a control nsCAR construct using a control sequence encoding a non-specific single-chain variable fragment to maintain identical molecular weights to replace the TNFR1 extracellular domain in anti-TNFα CAR (Figure 1A, nsCAR). A Myc tag was incorporated into the intermediate region of the construct to distinguish the presence of the TNFR1^30-212^ receptor domain in the anti-TNFα CAR, which was absent in the nsCAR. A HA tag was added following the MerTK domain to facilitate the detection of full-length anti-TNFα CAR and nsCAR expression (Figure 1A). To achieve macrophage-specific targeting and improve AAV-mediated transduction efficiency of the anti-TNFα CAR plasmids into BMDMs, the anti-CD11b AAV2 capsid was genetically modified to incorporate a CD11b-targeting nanobody that specifically recognizes the CD11b surface marker on BMDMs (Figure 1B). Successful recombinant anti-CD11b AAV2 packaging was confirmed by western blotting (Figure S2A). Anti-CD11b AAV2 achieved a significantly higher transfection efficiency (~15%) in BMDMs than the unmodified AAV2 (2.74%) (Figure 1C). To validate CD11b-targeting specificity, the engineered anti-CD11b AAV2 was transduced *ex vivo* into CD11b-negative 293T cells, anti-CD11b AAV2 transduced CD11b-negative 293T cells were inefficient, demonstrating its selective tropism (Figure S2B). Subsequently, anti-TNFα CAR and nsCAR expression was confirmed using fluorescence western blot (Figure 1D). We also observed co-localization of CD11b and anti-TNFα CAR on the cell membranes of macrophages using immunofluorescence (Figure 1E, Figure S2C).

### Binding and internalization of TNF-α by anti-TNFα CAR-M

**Figure 2:**
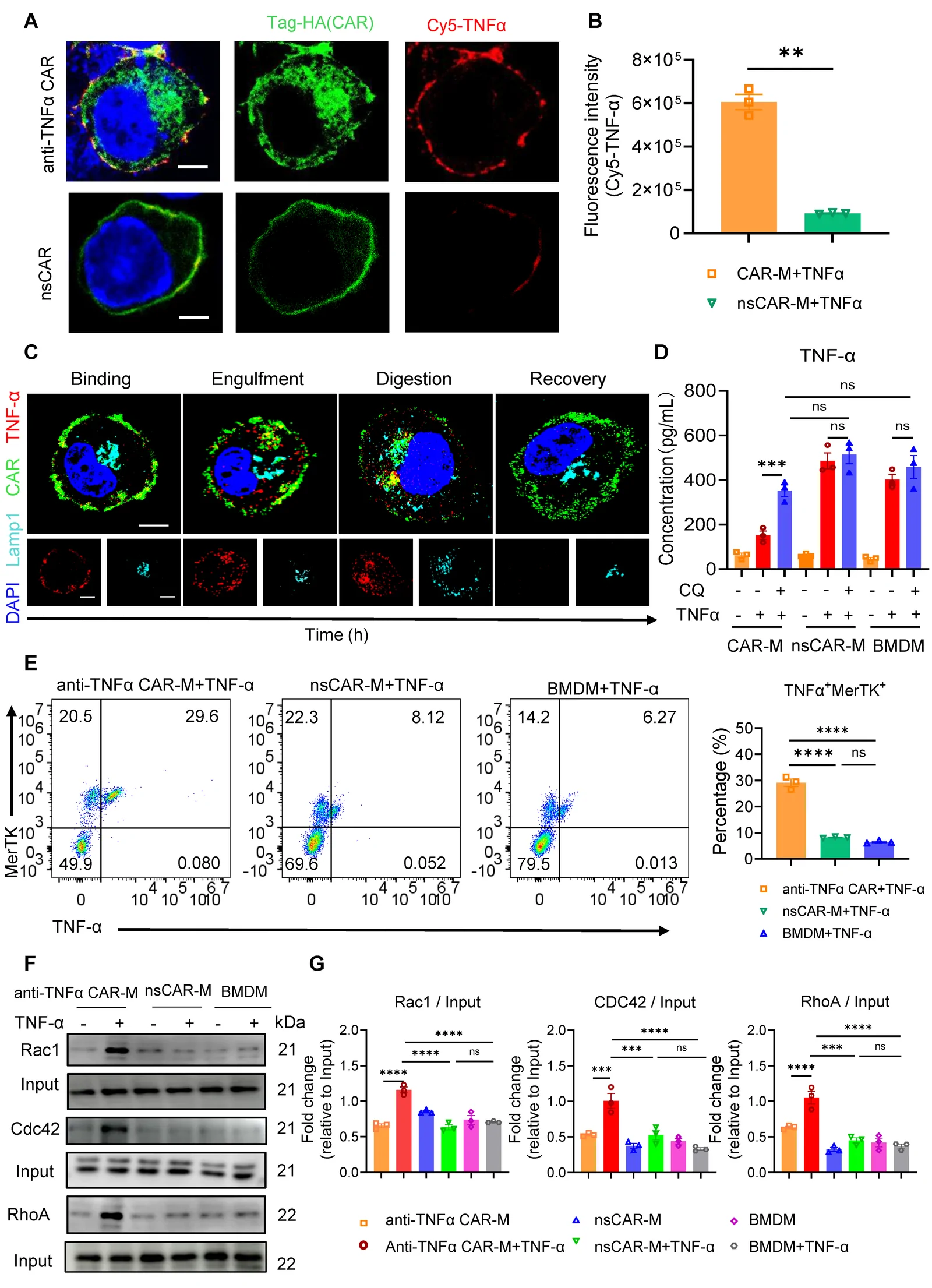
** The engulfment and digestion of TNF-α in Anti-TNFα CAR-M. (A)** Representative confocal images of the colocalization of Cy5-labeled-TNF-α (red) and CAR (green) on BMDMs. The BMDMs were transfected with the plasmids of anti-TNFα CAR-M, nsCAR-M for 72 h, after a 2 h-incubation with 20 ng/mL TNF-α, cells were labeled with anti-HA antibody. Scale bar represents 5 μm. **(B)** The fluorescence intensity of TNF-α and CAR in (A) was quantified by ImageJ software. n = 3. Data are shown as mean ± S.E.M, and an unpaired T test with two tailed was used for statistical analysis.** (C)** Representative confocal images showing TNF-α uptake and degradation stages. CAR-M were treated with 20 ng/mL Cy5-TNF-α for 12 h and washed with fresh medium after incubation, TNF-α and LAMP1 in CAR-M were stained with respective antibodies at different times and imaged by confocal microscopy. Scale bar represents 5 μm. **(D)** TNF-α levels in the culture supernatants were measured by ELISA following CQ treatment. Each symbol represents one independent biological replicate, and data are shown as mean ± SEM. n = 3. **(E)** Flow cytometric measurement of MerTK and TNF-α in anti-TNFα CAR-M or nsCAR-M. The BMDMs were transfected with the plasmids of anti-TNFα CAR-M, nsCAR-M for 72 h, after a 2 h-incubation with 20 ng/mL TNF-α, cells were stained with PE-labeled anti-MerTK antibody and APC-labeled TNF-α antibody and assessed by flow cytometry at an MOI of 200. **(F)** The activation of Rac1, Cdc42 and RhoA. Rac1, Cdc42 and RhoA were pulled down from the lysates of Anti-TNFα-CAR-M, nsCAR-M, and BMDM treated with or without TNF-α for 24 h at an MOI of 200, respectively. The samples were then subjected to Western blotting. Input Rac1, Cdc42 and RhoA were used as controls. **(G)** Quantitative analysis of the results shown in **(F)** using ImageJ software. n = 3. Data are shown as mean ± S.E.M, and one-way ANOVA was used for statistical analysis.

To validate the functional capacity of anti-TNFα CAR-M to bind and phagocytose TNF-α, Cy5-labeled TNF-α was added to anti-TNFα CAR-M cultures, and ICC analysis confirmed anti-TNFα CAR expression on BMDMs and its co-localization with TNF-α while TNF-α binding was hardly detected on the surface of nsCAR-transduced BMDMs (Figure 2A-B). To exclude the possibility that the enhanced TNF-α signal in CAR-M was mainly caused by endogenous macrophage TNFR1-mediated binding or uptake, we performed an additional TNFR1 blockade experiment. BMDMs were pretreated with an anti-TNFR1 blocking antibody before CAR or nsCAR transfection, followed by incubation with Cy5-labeled TNF-α. Under endogenous TNFR1 blockade, although both anti-TNFα CAR-M and nsCAR-M showed decreased fluorescence intensity of Cy5-labeled TNF-α to some extent, anti-TNFα CAR-M still showed a markedly stronger Cy5-TNF-α signal than nsCAR-M, whereas nsCAR-M exhibited only weak basal fluorescence (Figure S3). These results indicate that the enhanced TNF-α association in anti-TNFα CAR-M was mainly mediated by the engineered CAR structure rather than endogenous TNFR1 expressed by macrophages. Therefore, our results indicated that anti-TNFα CAR mediated main TNF-α binding. To further investigate the phagocytic and digestion process of TNF-α in anti-TNFα CAR-M, we performed time-course ICC study. The results showed that TNF-α firstly bind to anti-TNFα CAR expressed on the surface of BMDMs, then the complex of TNF-α and anti-TNFα CAR was internalized by BMDMs with the appearance of phagocytic vesicles. The internalized TNF-α was then colocalized with the lysosomal marker LAMP1, suggesting that phagocytosed TNF-α might be digested through the lysosomal pathway (Figure 2C-D). To minimize potential interference of endogenous TNF-α, before adding exogenous Cy5-labeled TNF-α, the original culture medium was removed, cells were changed into fresh medium, and the cells were washed three times with sterile PBS to reduce residual endogenous TNF-α present in the culture system. In addition, we measured supernatant TNF-α of CAR-M, nsCAR-M and BMDMs by ELISA before and after the addition of exogenous Cy5-TNF-α. The ELISA results showed that, in the absence of exogenous TNF-α, endogenous TNF-α levels in CAR-M, nsCAR-M, and BMDM cultures were notably lower than the concentration of exogenous TNF-α used in the uptake assay (Figure S4A). To exclude potential interference from endogenous TNF-α secreted by BMDMs, we also performed an additional endogenous TNF-α neutralization assay. After anti-TNFα CAR or nsCAR transfection, cells were pretreated with a neutralizing anti-TNFα antibody before exposure to exogenous Cy5-labeled TNF-α. After removing unbound antibodies, Cy5-TNF-α was added, and TNF-α association was evaluated based on Cy5 fluorescence (Figure S4B-C). Under this condition, CAR-M still displayed a stronger Cy5-TNFα-associated signal and time-dependent decrease in Cy5-TNF-α fluorescence, whereas nsCAR-M showed only weak Cy5-TNF-α association and no obvious degradation curve. These results suggest that the Cy5-TNF-α degradation kinetics observed in CAR-M were not mainly caused by endogenous TNF-α competition, but were dependent on CAR-mediated TNF-α recognition and clearance. To further verify the lysosome-dependent degradation of TNF-α by anti-TNFα CAR-M, BMDM, CAR-M, and nsCAR-M were pretreated with CQ before the addition of exogenous TNF-α, and TNF-α levels in the culture supernatants were determined by ELISA after 12 h. CAR-M lowered residual supernatant TNF-α to a level close to control cultures (Figure 2D), indicating efficient TNF-α clearance. In contrast, CQ treatment significantly increased the residual TNF-α level in the CAR-M supernatant, indicating that lysosomal pathway inhibition impaired TNF-α degradation in CAR-M, further supporting that CAR-M-mediated TNF-α clearance is dependent, at least in part, on lysosomal degradation. Further analysis showed that TNF-α was predominantly localized in MerTK^+^ BMDMs, even in the non-CAR-expressing control group (Figure 2E), indicating MerTK^+^ macrophage-mediated TNF-α uptake, which is consistent with previous report that MerTK⁺ macrophage is the major subpopulation resolving inflammation 3. Notably, the anti-TNFα CAR-treated group exhibited significantly enhanced TNF-α phagocytosis compared with other groups. Rho-family GTPases coordinate receptor-dependent actin remodeling during phagocytosis, with pathway-specific contributions from Rac1, Cdc42 and RhoA 26: the Rac1/Cdc42-associated FcγR pathway which involves membrane protrusions, and the RhoA-dependent complement receptor 3 pathway which relies on cytoskeletal rearrangement. Our pull-down assays demonstrated significant activation of Rac1, Cdc42 and RhoA were activated selectively in anti-TNFα CAR-M rather than nsCAR-M (Figure 2F-G), suggesting that both pathways contribute to TNF-α phagocytosis in anti-TNFα CAR-M.

### Anti-TNFα CAR-M remodels the inflammatory microenvironment and promotes inflammation-resolving macrophage polarization

**Figure 3:**
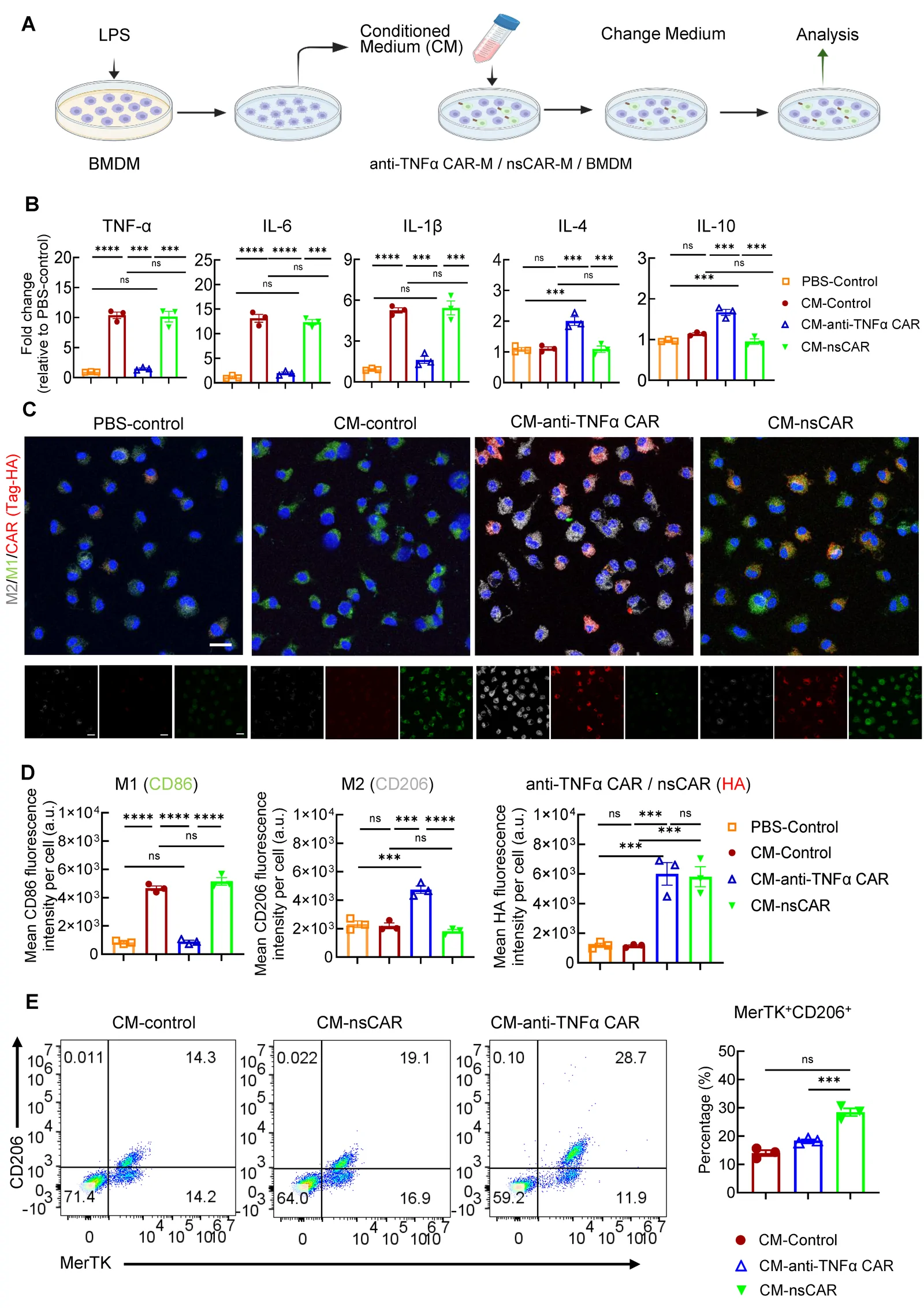
** CAR-M attenuates inflammatory microenvironment and polarizes macrophages toward MerTK^+^CD206^+^ phenotype. (A)** Cartoon depicting the experimental paradigm used to investigate the effect of CAR-M on LPS-triggered inflammatory responses in BMDMs. BMDMs were stimulated with 1 μg/mL LPS for 12 h to generate conditioned medium (CM). This CM was then applied to anti-TNFα CAR-M, nsCAR-M, and BMDMs for 6 h. Cytokine levels in supernatants and cellular phenotypes were analyzed, with controls including PBS-treated BMDMs (PBS-Control), CM-treated BMDMs (CM-Control), CM-treated anti-TNFα CAR-M (CM-anti-TNFα CAR), and CM-treated nsCAR-M (CM-nsCAR). **(B)** The levels of TNF-α, IL-6, IL-1β, IL-4, and IL-10 in anti-TNFα CAR-treated BMDMs. The BMDMs were transfected with anti-TNFα CAR-M, nsCAR-M or control for 72 h, after a 24 h-incubation with LPS-induced CM the supernatant of BMDMs were detected by ELISA (relative to BMDMs control). n = 3. Data are shown as mean ± S.E.M, and one-way ANOVA was used for statistical analysis. **(C)** Representative confocal image of expression of M1 (CD86, green), M2 (CD206, grey) marker and CAR (red) in anti-TNFα CAR-Ms (CM-CAR), nsCAR-Ms (CM-nsCAR), and BMDMs (PBS-control and CM-control). The BMDMs were transfected with anti-TNFα CAR-M, nsCAR-M for 72 h, after a 24 h-incubation with LPS- CM, cells were stained with anti-CD86, anti-CD206, and anti-HA. **(D)** Relative mean fluorescence levels of CD86, CD206, CAR in (C) were quantified in ImageJ. n = 3. Data are shown as mean ± S.E.M, and one-way ANOVA was used for statistical analysis. **(E)** Flow cytometry analysis of MerTK and CD206 in BMDM-treated with anti-TNFα CAR, nsCAR in the presence of LPS-CM. The BMDMs were transfected with anti-TNFα CAR-M, nsCAR-M for 72 h, after a 24 h-incubation with LPS-CM, cells were stained with PE-labeled anti-MerTK antibody and FITC-labeled anti-CD206 antibody, and assessed by flow cytometry. n = 3. Data are shown as mean ± S.E.M, and one-way ANOVA was used for statistical analysis.

**Figure 4:**
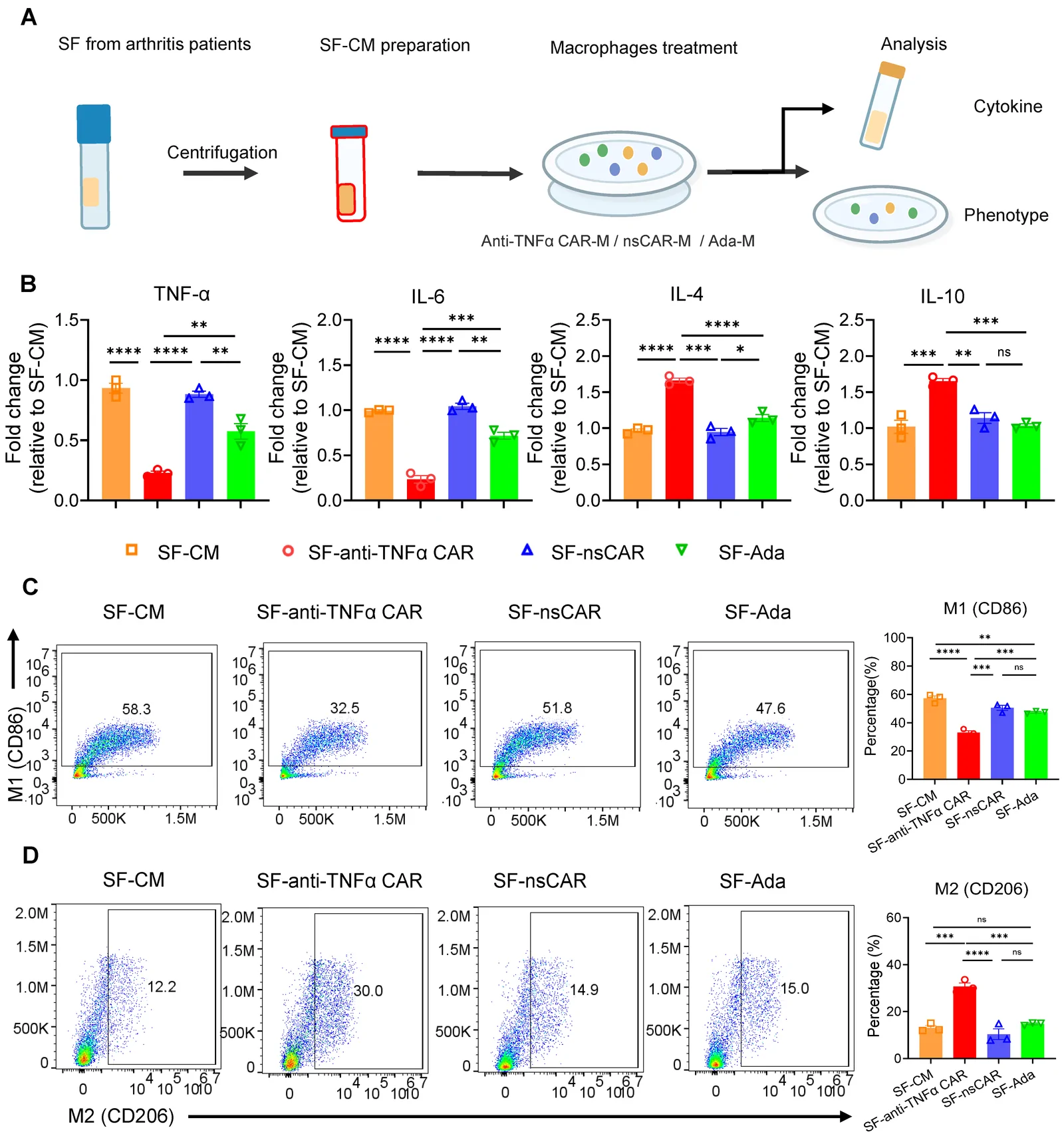
** CAR-M remodels macrophage inflammatory responses under arthritis patient synovial fluid-conditioned medium stimulation. (A)** Schematic of the experimental design used to investigate the effects of CAR-M, nsCAR-M, and adalimumab-treated macrophage (Ada-M) on the SF-CM derived from arthritis patients to mimic the inflammatory joint microenvironment. **(B)** Levels of inflammatory and anti-inflammatory cytokines, including TNF-α, IL-6, IL-4, and IL-10, in the culture supernatants were quantified after treatment. n = 3. Data are shown as mean ± S.E.M, and one-way ANOVA was used for statistical analysis. **(C, D)** Flow cytometry analysis and quantification of M1-like (CD86) and M2-like (CD206) macrophage populations after SF-CM stimulation. n = 3. Data are presented as mean ± S.E.M.

Inflammatory microenvironment enhancement plays a critical role in RA pathogenesis 27. To evaluate the immunomodulatory effects of anti-TNFα CAR-M on inflammatory microenvironment, we firstly analyzed cytokine production by macrophage model with LPS treatment and then added the conditioned medium (CM) from the macrophages treated with LPS to the cultures of anti-TNFα CAR-M or nsCAR-M (Figure 3A). Anti-TNFα CAR-M markedly decreased TNF-α, IL-6, and IL-1β, whereas IL-10 and IL-4 were concomitantly increased in CM of anti-TNFα CAR-M relative to nsCAR-M and CM-Control (Figure 3B). Traditionally, macrophages are commonly described along an M1-like inflammatory to M2-like resolving spectrum, which is typically marked by CD86 and CD206, respectively. Within the M2 population, MerTK⁺CD206⁺ macrophages constitute the principal subset responsible for the resolution of inflammation in RA 3. IL-10 promotes MerTK induction in human macrophages 28, whereas IL-4 promotes CD206 expression 29. Therefore, we performed immunofluorescence staining to detect macrophage markers including CD86, CD206, and MerTK. The results showed that, compared with the CM treated nsCAR-M, CM treated anti-TNFα CAR-M exhibited a significantly increased mean fluorescence intensity in CD206^+^ M2 macrophages while a marked decrease in M1 macrophages (Figure 3C-D). In anti-TNFα CAR-M groups, MerTK expression was moderately elevated compared to nsCAR-M controls, suggesting that anti-TNFα CAR-mediated anti-inflammatory effects may concurrently stimulate endogenous MerTK production in macrophages (Figure 3E). Additionally, the population of MerTK⁺CD206^+^ macrophages showed marked elevation in anti-TNFα CAR-M (Figure 3E), indicating that anti-TNFα CAR expression promoted macrophage polarization toward MerTK⁺CD206^+^ phenotype. Anti-TNFα CAR activation by the binding of TNF-α to TNF-R1 suppressed the production of pro-inflammation cytokines and enhanced IL-4 and IL-10 expression, ultimately increasing expression of CD206 and MerTK. In contrast, nsCAR-M lacking TNF-R1 failed to activate the MerTK pathway or suppress inflammation. To further enhance the clinical relevance of the anti-inflammatory effect of CAR-M cells, we used synovial fluid from patients with arthritis as a disease-relevant conditioned medium (SF-CM) to mimic the complex inflammatory milieu of arthritic joints (Figure 4A). Because patient-derived synovial fluid contains multiple inflammatory mediators in addition to TNF-α, we then examined whether the macrophage-remodeling effect of CAR-M was associated with other inflammatory mediators, or merely attributable to TNF-α neutralization or involved CAR-mediated intracellular signaling. Adalimumab was included as a clinically used TNF-α neutralization control under SF-CM stimulation. Compared with nsCAR-M and adalimumab-treated CAR-M, anti-TNFα CAR-M more effectively lowered TNF-α and IL-6 levels and increased IL-4 and IL-10 (Figure 4B). CAR-M cells also shifted the macrophage profile from M1-like toward M2-like (Figure 4C-D).

### Anti-TNFα CAR-M prevents inflammation by multiple signaling pathways

**Figure 5:**
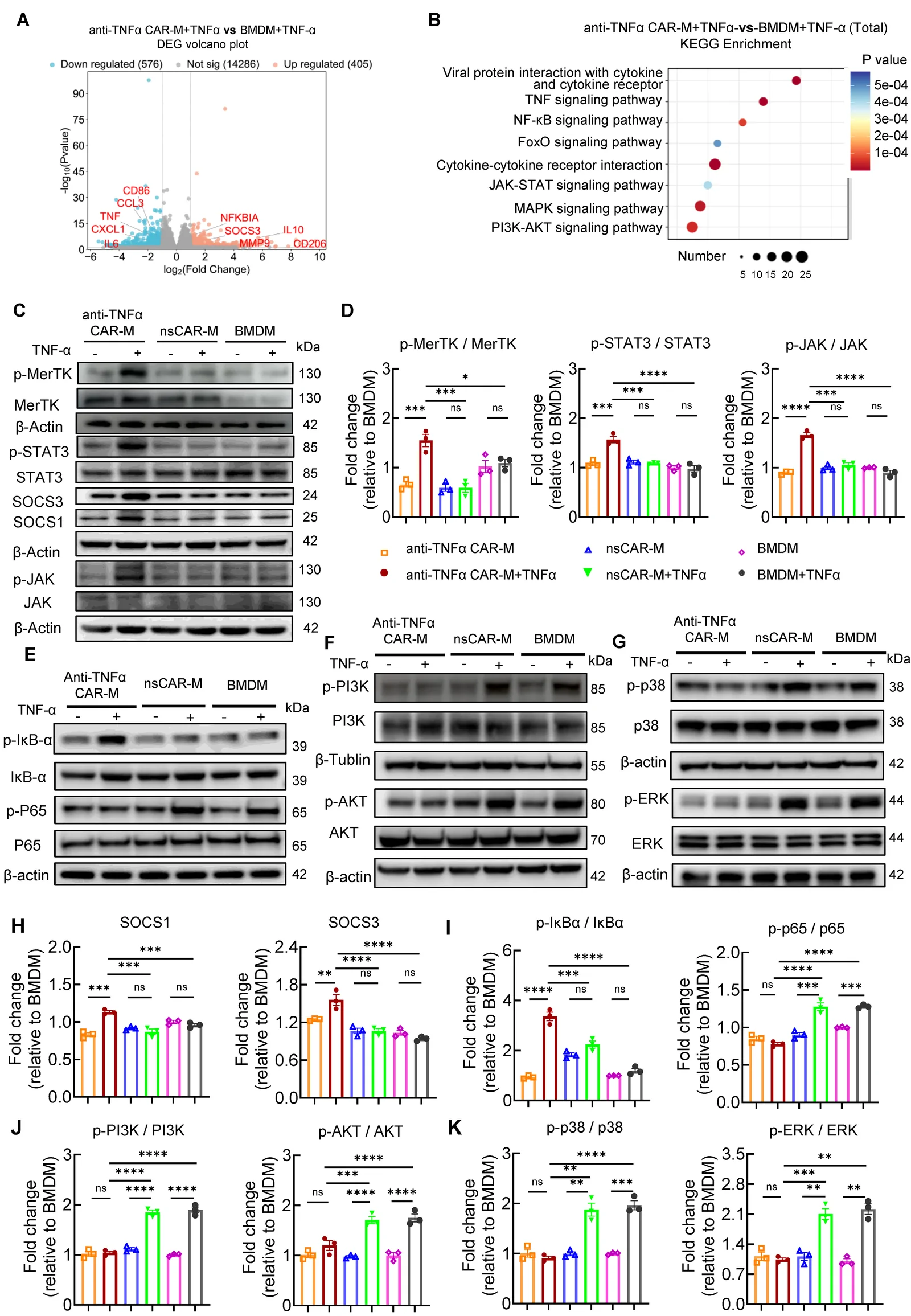
** Anti-TNFα CAR-M prevents inflammation via multiple anti-inflammatory pathways. (A)** A volcano plot illustrating differentially regulated gene (DEGs) expression from RNA-seq analysis between BMDMs and anti-TNFα CAR-treated BMDMs in the presence of TNF-α. BMDMs and anti-TNFα CAR-M were treated with 20 ng/mL TNF-α for 12 hours, followed by cell collection and RNA extraction for RNA-seq analysis. The adjusted p values were obtained with a two-sided Wilcoxon test. Not sig, not significant (p-value > 0.05 and |log_2_FC| < 1); Sig, significant (p-value < 0.05 and |log_2_FC| > 1); up and down indicate the intersecting DEGs significantly up- and down- regulated in anti-TNFα CAR-treated group (p-value > 0.05), respectively. n = 4. **(B)** KEGG pathway enrichment in anti-TNFα CAR-Ms vs BMDMs in the presence of TNF-α.** (C)** Key proteins from MerTK downstream signaling pathway were subjected to western blotting. Anti-TNFα CAR-Ms, nsCAR-Ms and BMDMs were stimulated with LPS-conditioned medium for 24 hours, followed by cytolysis and total protein extraction. Target proteins (including p-MERTK, MERTK, p-STAT3, STAT3, p-JAK, JAK, SOCS1, SOCS3) were detected using their respective specific antibodies.** (D-H)** Relative protein levels in **(C)** quantified in ImageJ. n = 3. Data are shown as mean ± S.E.M and statistical analysis used one-way ANOVA with Tukey’s post hoc test.** (E-G)** Key proteins from NF-κB **(E)**, PI3K-AKT **(F)**, MAPK **(G)** pathways were subjected to western blotting. **(I-K)** Relative protein levels in** (E-G)** quantified in ImageJ. n = 3. Data are shown as mean ± S.E.M, and statistical analysis used one-way ANOVA with Tukey’s post hoc test.

To define how anti-TNFα CAR limited inflammation, we performed RNA-seq analysis on anti-TNFα CAR-M and BMDMs under TNF-α stimulation to identify differentially expressed genes (DEGs) (Figure 5A). The results revealed ant-inflammation genes such as MerTK, SOCS3, IL-10, MMP9, NFKBIA were upregulated, while pro-inflammation and chemokines genes such as CD86, IL-6, TNF, CCL2, CCL3, CXCL1, CXCL2 along with other genes *VEGFA, TGFBR1* were markedly downregulated in anti-TNFα CAR-M relative to BMDMs control in the presence of TNF-α (Figure 5A). To exclude potential non-specific transcriptional effects caused by the CAR scaffold or intracellular MerTK signaling module, we applied nsCAR-M + TNF-α group as an additional qPCR validation control for selected RNA-seq-derived DEGs. MerTK expression was comparable between nsCAR-M and CAR-M cells, suggesting a similar expression of the MerTK^+^ containing CAR signaling module (Figure S5A). However, upon TNF-α stimulation, CAR-M cells showed a transcriptional profile distinct from those of both nsCAR-M and BMDMs. IL-10 and MMP9 were markedly increased in CAR-M + TNF-α (Figure S5B), whereas inflammatory chemokine genes, including CCL2, CCL3, CXCL1 and CXCL2, were significantly decreased (Figure S5C). In contrast, nsCAR-M + TNF-α did not reproduce these transcriptional changes. These results support that the RNA-seq-identified macrophage remodeling signature was not simply caused by non-specific CAR module expression, but required TNF-α recognition by the full anti-TNFα CAR. KEGG analysis further demonstrated that these DEGs have functions related to NF-κB, cytokine-cytokine receptor interactions, JAK-STAT, MAPK, and PI3K-AKT pathways (Figure 5B), which are involved in the progression of RA 30-32. Subsequently, key proteins from these pathways and the MerTK signaling pathway were examined using western blotting. The results showed that compared to nsCAR-M + TNF-α and BMDM + TNF-α groups, the phosphorylation levels of MerTK, STAT3, and JAK, and the expression levels of SOCS1 and SOCS3 in anti-TNFα CAR-M group was markedly increased (Figure 5C-D, H). Notably, although the hyperactivation of JAK-STAT signaling is generally linked to RA progression, the simultaneous upregulation of SOCS1, SOCS3, and IL-10 suggests that elevated p-JAK and p-STAT3 levels reflect the activation of a MerTK-driven anti-inflammatory feedback loop rather than a pro-inflammatory response. Previous reports have demonstrated that the downstream signaling pathways of MerTK are activated only when two adjacent MerTK receptors simultaneously bind to their ligands 33. TNF-α naturally exists as a homotrimer and contains three TNFR1-interacting sites, and the binding of two or three CARs on macrophages to the same TNF-α was readily induced, thereby triggering MerTK phosphorylation, actin remodeling and TNF-α uptake. Meanwhile, in NF-κB signaling pathway, the phosphorylation level of protein IκBα was notably elevated while the level of p-P65 was significantly reduced in anti-TNFα CAR-M relative to other groups (Figure 5E, I). In PI3K-AKT signaling pathway, there was a pronounced decrease in the phosphorylation level of PI3K and AKT (Figure 5F, J), and in MAPK signaling pathway, the phosphorylation of p38 and p-ERK were significantly reduced in anti-TNFα CAR-M relative to other groups (Figure 5G, K).

### Anti-TNFα CAR therapy alleviates RA pathology in CIA mice

**Figure 6:**
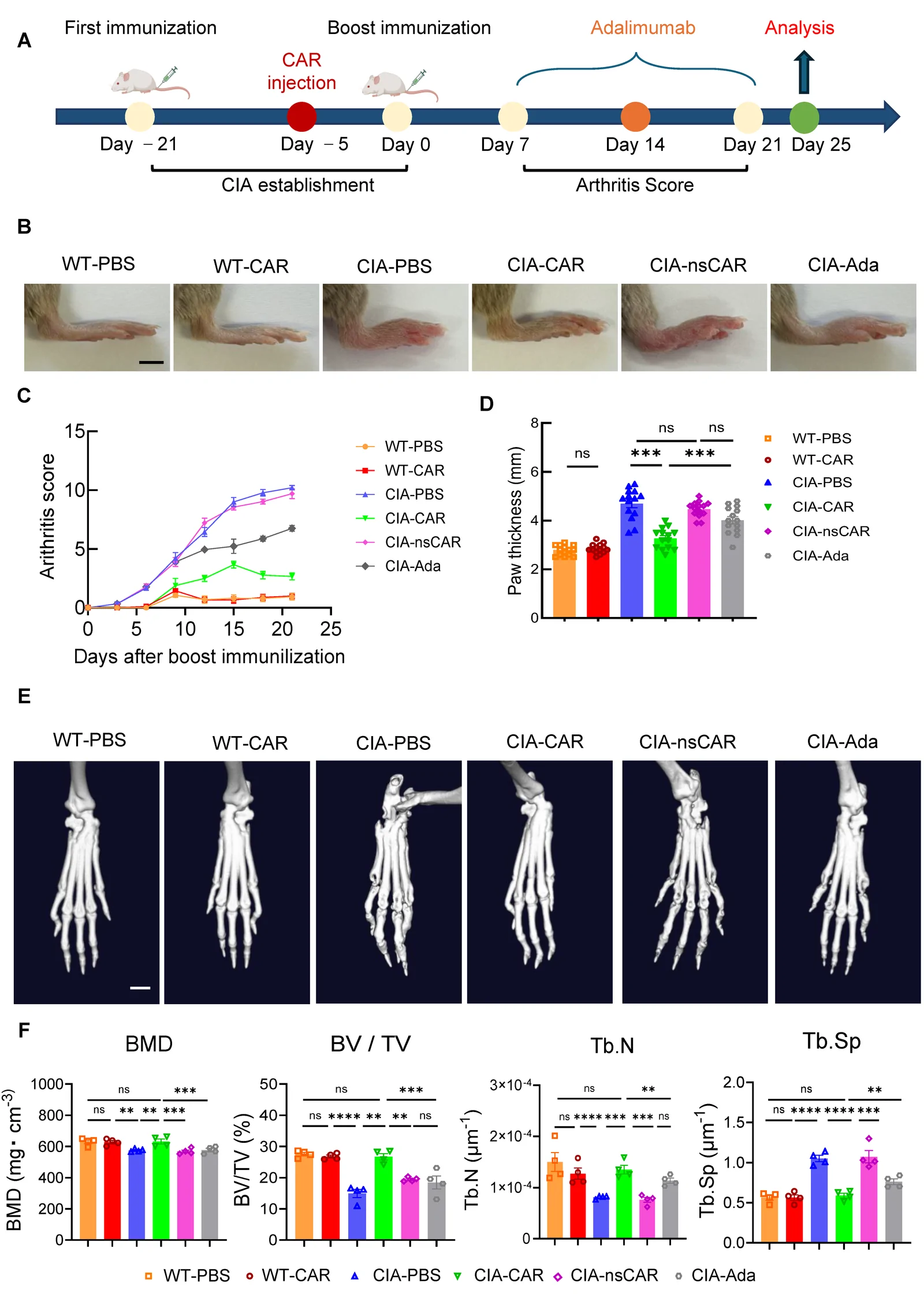
** Anti-TNFα-CAR (CAR) alleviates RA pathology in CIA mice. (A)** Experimental protocol for CIA induction and downstream evaluation of therapeutic effects of anti-TNFα CAR. CIA was established in mice using the following protocol: primary immunization with chicken II collagen was performed on day -21 via subcutaneous injection, and boost immunization was given on day 0. On day -5, AAV-delivered the plasmid of anti-TNFα CAR (CAR), nsCAR or PBS was administered intra-articularly to bilateral ankle joints. On Day 7 after the booster immunization, the biologic-control CIA mice received intraperitoneal adalimumab at 1.5 mg/kg once every 3 days until the experimental endpoint. Arthritis scores were recorded every three days during the peak inflammatory phase (day 7-21). On day 25, micro-CT experiments, inflammatory cytokine quantification, and histopathological examination of the ankle joints of mice were conducted. **(B)** Representative images of hind paws from different treatment groups on day 25. Scale bar indicates 2 mm. **(C)** Arthritis scores in CIA mice following CAR treatment (n = 7). Arthritis scores were monitored every 3 days for 21 days.** (D)** Quantification of hind paw thickness in** (B)** was performed using the Avatar analysis software (V.1.6.5.3) (n = 14 hind paws, including measurements of both the left and right hind paws from seven mice per group). Data are presented as mean ± S.E.M, and one-way ANOVA was used for statistical analysis.** (E)** Representative 3D micro-CT reconstruction of the ankle joints.n = 4. Scale bar indicates 1 mm. **(F)** Quantitation of BMD, BV/TV, Tb.N, and Tb.Sp in **(E)** was performed using Avatar (V.1.6.5.3) and CTan. n = 4. Data are shown as mean ± S.E.M, and one-way ANOVA was used for statistical analysis.

To test *in vivo* efficacy and safety, CIA mice were randomly assigned to different treatment groups after disease induction. AAV-CAR was locally administered before booster immunization to generate the CIA-CAR treatment group, whereas CIA mice that received PBS served as the disease control group. Age-matched wild-type mice that received the same dose of AAV-CAR were included as biosafety controls (WT-CAR). In parallel, CIA mice treated with adalimumab were used as clinically relevant biological treatment controls (CIA-Ada). Disease progression and therapeutic responses were monitored longitudinally after booster immunizations (Figure 6A). Arthritis severity was assessed using the arthritis score, which is based on the criteria of redness and swelling of the hind paws from the ankle, tarsal, and metatarsal joints to the digits, using paw swelling scores, micro-CT imaging, and histopathological analyses. The results showed that adalimumab treatment exhibited a marked therapeutic effect on paw swelling, arthritis scores, and joint erosion compared to the PBS control. However, anti-TNFα CAR treatment reduced paw swelling and arthritis scores more effectively than treatment of nsCAR and CIA-Ada, with disease scores approaching those of healthy controls by day 21 (Figure 6B-D). Microarchitectural analysis of the ankle joint was performed on day 25 using micro-CT. Compared with CIA-nsCAR or CIA-Ada mice, anti-TNFα CAR-treated CIA mice exhibited minimal joint erosion (Figure 6E-F). Consistently, the decreases in BMD, BV/TV and Tb.N, as well as the increase in Tb.Sp, observed in CIA-PBS mice were largely reversed after anti-TNFα CAR treatment. These micro-CT results indicate that anti-TNFα CAR treatment effectively preserved periarticular bone microarchitecture and exerted a strong bone-protective effect in CIA mice.

### Anti-TNFα CAR reduces systemic and local inflammation in CIA mice

**Figure 7:**
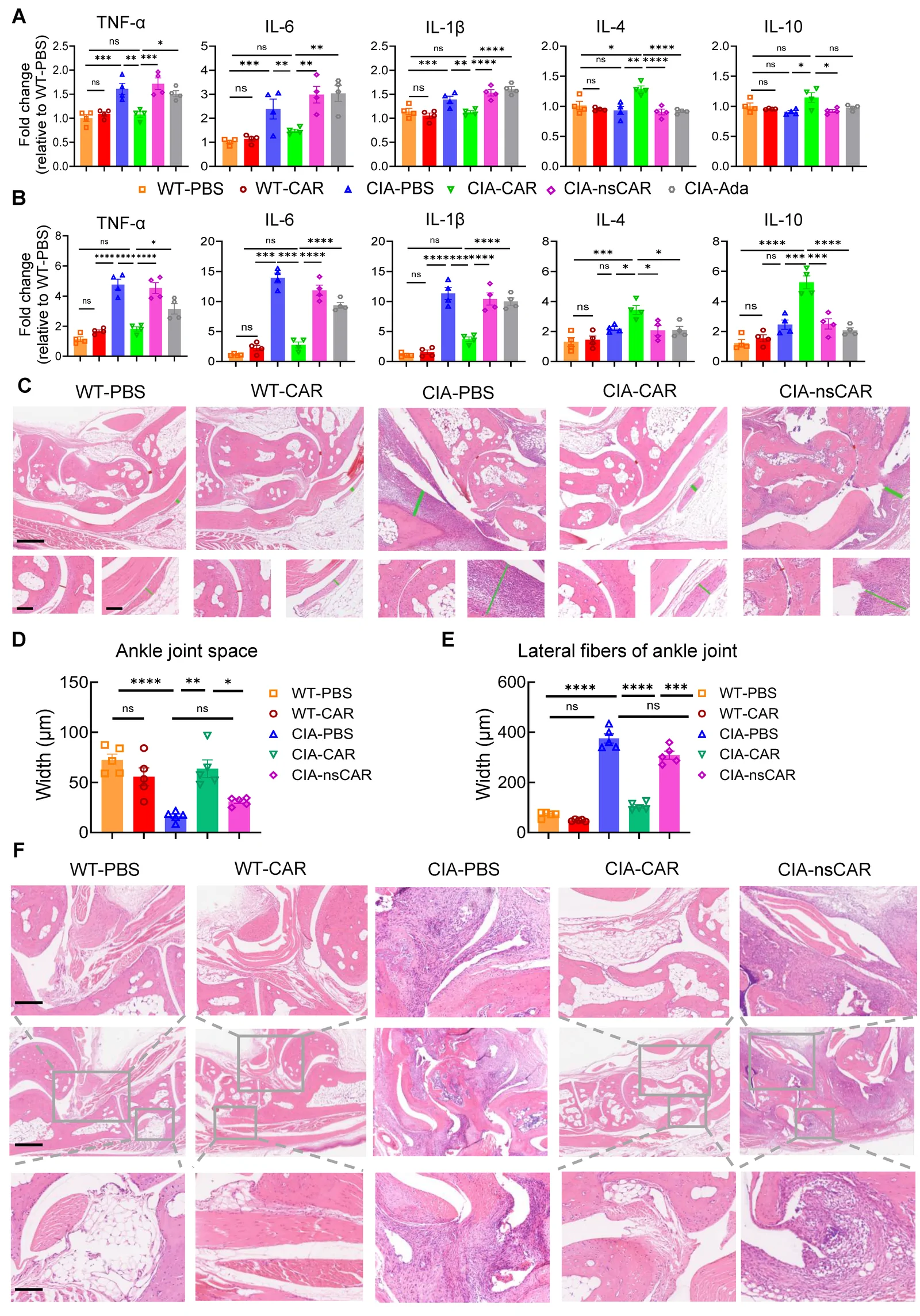
** Anti-TNFα CAR resolves inflammation in CIA mice. (A)** The levels of TNF-α, IL-6, IL-1β, IL-4, and IL-10 in the serum of six mouse groups on day 14 were analyzed by ELISA (relative to WT-PBS). WT-PBS (healthy control), WT-CAR (anti-TNFα CAR-treated healthy controls), CIA-PBS (diseased controls), CIA-CAR (anti-TNFα CAR-treated diseased controls), CIA-nsCAR (nsCAR-treated diseased controls), and CIA-Ada (adalimumab-treated diseased controls) n = 4. **(B)** TNF-α, IL-6, IL-1β, IL-4, and IL-10 levels in the ankle joint area of six mouse groups on Day 14 were analyzed. n = 4. Data are shown as mean ± S.E.M, and a one-way ANOVA was used for statistical analysis.** (C)** Representative images of H&E staining of ankle joint sections. Red lines and green lines indicate the width of ankle joint space and lateral fibers of the ankle joint in WT-PBS, WT-CAR, CIA-PBS, CIA-CAR, and CIA-nsCAR. Scale bar represents 400 μm. **(D-E)** Quantification of the ankle joint space width **(D)** and lateral fibers width **(E)** in **(C)** by K-Viewer. n = 5 Data are shown as mean ± S.E.M, and a one-way ANOVA was used for statistical analysis.** (F)** Representative H&E-stained ankle joint sections for assessment of synovial inflammation. Scale bar represents 400 μm. The boxes display unilateral or bilateral fibroadenoma formation (up, scale bar represents 200 μm) and inflammatory cell infiltration status in the synovial space (down, scale bar represents 100 μm).

**Figure 8:**
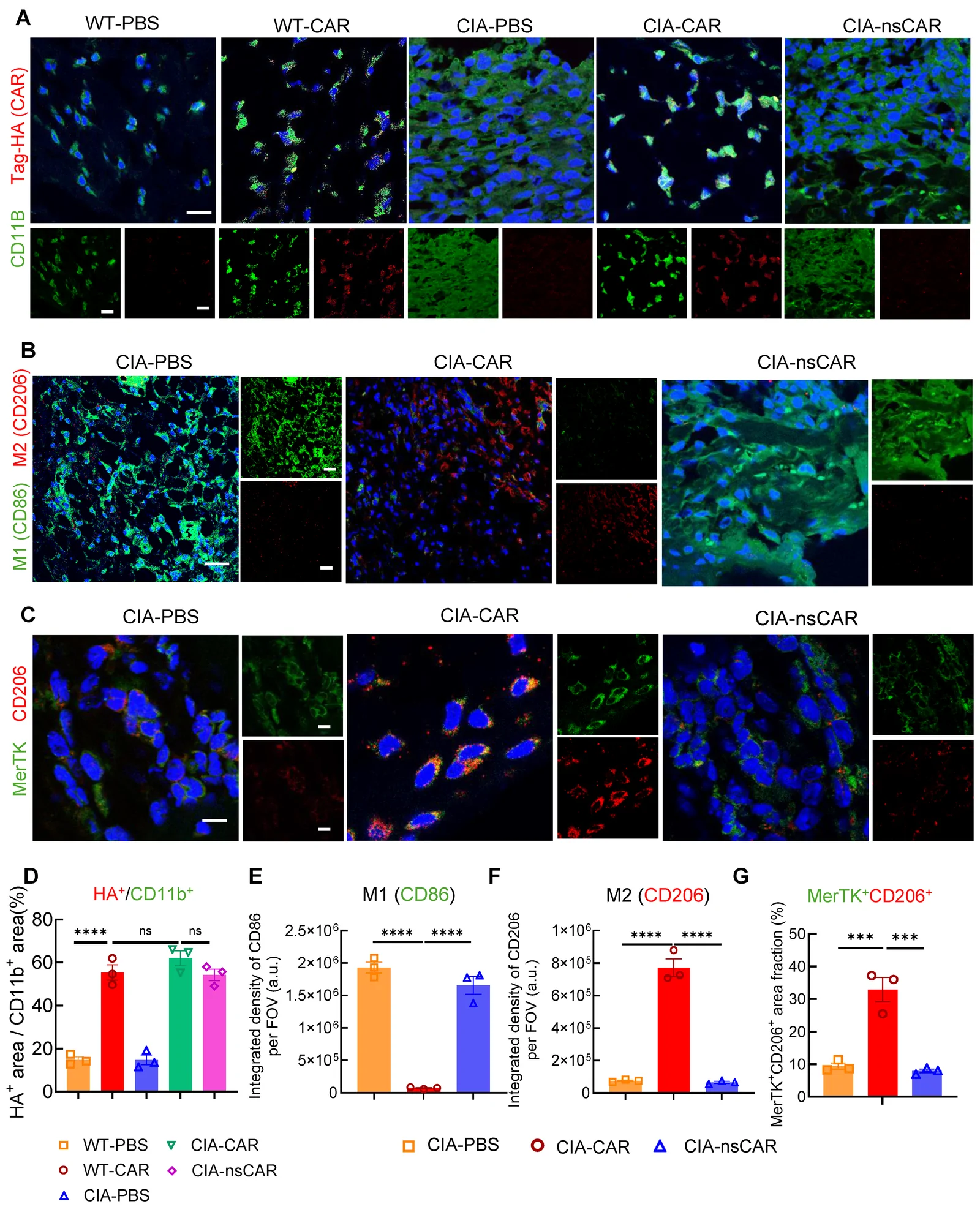
** Anti-TNFα CAR polarizes macrophages toward MerTK^+^CD206^+^ phenotype in the joint area of CIA mice. (A)** Representative confocal image of CAR expression on macrophages in the ankle joint area of five groups. The levels of CAR (tag-HA, red) on macrophages (CD11b, green) in the ankle joint were detected by IHC. Scale bar represents 25 μm. **(B)** Representative confocal image of M1 (CD86, green) and M2 (CD206, red) macrophage marker expression in the ankle joint. **(C)** Representative confocal image of MerTK (green) and CD206 (red) expression in the ankle joint area of three groups. Scale bar represents 50 μm. **(D)** Relative levels of CAR (HA-tag) of macrophage (CD11b) in** (A)**. **(E)** M1 (CD86) and **(F)** M2 (CD206) in **(B)**, **(G)** MerTK and CD206 in **(C)** were quantified in ImageJ. n = 3. Data are shown as mean ± S.E.M, and one-way ANOVA was used for statistical analysis.

To assess whether CAR-M also suppressed inflammation *in vivo*, we firstly examined TNF-α cytokine levels and macrophage states in CIA ankle joints. Our results revealed a marked elevation in TNF-α levels accompanied by a significant increase in pro-inflammatory M1 macrophages in CIA mice (Figure S6A-C). We measured cytokine levels in CIA mice (representing the disease groups) on days 7 and 14. By day 7, compared to the PBS-treated CIA controls (CIA-PBS), the anti-TNFα CAR-CIA group (CIA-CAR) showed non-significant reductions in pro-inflammatory cytokines (TNF-α, IL-6, IL-1β). This transient effect may reflect insufficient anti-TNFα CAR expression levels during the early phase of AAV-mediated gene delivery, potentially below the therapeutic threshold required for robust anti-inflammatory responses (Figure S7A). By day 14, these reductions were significant (Figure 7A). The qPCR analyses of ankle joint tissues were consistent with this finding (Figure 7B). Moreover, our results of H&E-stained joint sections showed that anti-TNFα CAR, but not nsCAR significantly reduced extensive fibrotic encapsulation and inflammatory infiltration in the joint cavity of CIA mice (Figure 7C). Furthermore, osteoclasts were detected together with a narrower width of the ankle joint cavity space in the CIA-PBS- and nsCAR-treated mice (Figure S7B), indicating that inflammation caused significant bone erosion and bone damage to the ankle joint. In contrast, anti-TNFα CAR treatment markedly rescued these symptoms (Figure 7C-F). Although the anti-TNFα CAR constructs were locally administered by intra-articular AAV injection, we further evaluated its biosafety because locally delivered vectors may still enter systemic circulation. Therefore, major organs, including the heart, liver, spleen, lungs, and kidneys, were collected from WT-PBS and WT-CAR mice for H&E staining, and serum biochemical parameters were analyzed. Histological examination revealed no obvious pathological abnormalities, inflammatory infiltration, or tissue damage in the major organs (Figure S8A). Serum biochemical analyses consistently revealed no abnormal changes in CREA, CK-MB, TBIL, DBIL, ALP, or AST levels in WT-CAR mice relative to those in WT PBS controls (Figure S8B). These findings indicate that local delivery of the anti-TNFα CAR constructs did not induce detectable systemic organ toxicity under the tested conditions. Immunofluorescence staining of joint sections confirmed anti-TNFα CAR and nsCAR was transduced into CD11b^+^ cells in ankle joints (Figure 8A, D). Because CD11b-expressing immune cells in inflamed mouse joints are not limited to macrophages but may also include neutrophils, dendritic cells, and other myeloid subsets, we performed multicolor immunofluorescence staining to evaluate whether the therapeutic vector signal was distributed among these non-macrophage CD11b^+^ populations.

The CAR signal showed minimal overlap with Ly6C/G^+^ neutrophils (Figure S9A) or CD11c^+^ dendritic cells (Figure S9B), whereas strong co-localization was observed with CD11b^+^F4/80^+^ macrophage lineage cells in arthritic joint tissues (Figure S9C). These data indicated that within the inflamed joint microenvironment, the CAR signal was mainly expressed in CD11b^+^F4/80^+^ macrophage-lineage cells rather than in other CD11b-expressing immune subsets.

A shift from M1 (CD86^+^) to M2 (CD206^+^) macrophages was observed in CAR-treated joints (Figure 8B, E-F), but not in the nsCAR-treated control. Consistent with these findings, anti-TNFα CAR-treated group showed significantly elevated levels of both MerTK and CD206 (Figure 8C, G), whereas nsCAR controls showed only MerTK upregulation without significant CD206 induction.

## Discussion

RA progression arises from the coordinated interactions between immune cells, stromal elements, and cytokine circuits, which collectively drive synovial inflammation, cartilage injury, and bone loss 34. TNF-α inhibitors have transformed RA treatment, yet approximately one third of treated patients have an inadequate or suboptimal response 7. Flares are also frequently accompanied by dose reduction or treatment cessation, including the tapering or withdrawal of adalimumab 35,36. Taken together, these limitations highlight a need to suppress pathogenic cytokines while re-establishing regulatory activity in the synovial compartment, without detracting from the clinical importance of TNF-α inhibition. Synovial macrophages are closely associated with active disease and sustained remission, and MerTK-expressing macrophages have been linked to restoration of the synovial lining barrier and joint homeostasis 3. The protective contribution of MerTK-dependent efferocytosis is also supported by experimental models of arthritis 16.

The anti-TNFα chimeric antigen receptor macrophage (CAR-M) developed here was designed to address both components of this problem. Its TNFR1-derived extracellular domain recognizes TNF-α, whereas its intracellular MerTK domain is intended to couple ligand engagement to phagocytic and immunoregulatory signaling. Although both forms of TNF interact with TNFR1 and TNFR2, soluble TNF efficiently stimulates TNFR1, whereas strong TNFR2 activation is more closely linked to transmembrane ligand 22. Selection of the TNFR1 recognition element therefore aligns with the aim of capturing soluble TNF-α in the extracellular space. Rather than functioning solely as a passive cytokine trap, this design treated the pathogenic mediator as a trigger for its removal and concomitant macrophage reprogramming.

Several findings support CAR-dependent TNF-α recognition and clearance. Anti-TNFα CAR-M showed greater TNF-α association than non-specific CAR macrophages, and this difference persisted after blockade of endogenous TNFR1 and after neutralization of macrophage-derived TNF-α. Time-course imaging showed internalization of the CAR–TNF-α complex and co-localization of internalized TNF-α with LAMP1-positive lysosomes. Chloroquine increased residual extracellular TNF-α, indicating that lysosomal degradation contributed to clearance. The activation of Rac1, Cdc42, and RhoA further implicates coordinated cytoskeletal remodeling and phagocytic machinery, consistent with the established roles of Rho-family GTPases in actin-dependent uptake 37. These data extend conventional TNF-α sequestration by demonstrating active cellular uptake and intracellular disposal of the targeted cytokine.

CAR engagement was also associated with a macrophage state enriched for MerTK and CD206, increased IL-4 and IL-10, and reduced TNF-α, IL-6 and IL-1β. MerTK signaling can promote the production of specialized pro-resolving mediators and support the restoration of tissue homeostasis 38. In the present system, non-specific CAR retained the MerTK-containing intracellular scaffold but lacked the TNFR1-derived recognition domain. Its inability to reproduce the transcriptional and phenotypic changes observed with the full anti-TNFα CAR indicates that scaffold expression alone was insufficient and that TNF-α recognition was required. Nevertheless, because this study did not include genetic deletion or selective inhibition of the CAR-associated MerTK module, the extent to which the individual downstream effects are directly caused by MerTK signaling remains to be determined.

The immunomodulatory activity of anti-TNFα CAR-M was observed in both LPS-CM and SF-CM derived from patients with arthritis. Synovial fluid contains multiple inflammatory mediators and can regulate stromal and immune-cell behavior through TNF and NF-κB-dependent mechanisms 39; it therefore provides a more disease-relevant challenge than stimulation with a single recombinant cytokine. Under synovial fluid-conditioned conditions, anti-TNFα CAR-M reduced TNF-α and IL-6, increased IL-4 and IL-10, and shifted the measured macrophage phenotype away from CD86 and toward CD206 more effectively than the adalimumab comparator used in this assay. Anti-TNF treatment has previously been associated with IL-10- and STAT3-dependent polarization of rheumatoid macrophages 40. IL-10 has broad anti-inflammatory activity 41 and contributes to MerTK induction in human macrophages 28. The current findings suggest that CAR-mediated TNF-α clearance and intracellular signaling can reinforce this regulatory cytokine environment.

However, CD86 and CD206 measurements should be interpreted as operational markers rather than as evidence of two fixed macrophage lineages. Macrophage activation occupies a multidimensional continuum, and consensus guidelines caution against equating limited marker panels with stable M1 or M2 identities 42. Accordingly, the combined increase in MerTK and CD206, the reduction in inflammatory mediators and chemokines, and the induction of IL-10 provided stronger evidence of a pro-resolving functional shift than CD206 expression alone. Validation of larger panels of primary human synovial macrophages with single-cell or spatial profiling is important for defining the precise state induced by the CAR.

Transcriptomic and protein analyses further showed that anti-TNFα CAR-M did not simply suppress all inflammatory signaling uniformly. The expression levels of IL-6, TNF-, CCL2, CCL3, CXCL1, and CXCL2 decreased, whereas those of IL-10, MMP9, NFKBIA, MerTK, and SOCS3 increased. At the protein level, phosphorylation of p65, PI3K, AKT, p38 and ERK decreased, consistent with attenuation of several pathways implicated in RA inflammation 43 and with reduced TNF-α signaling 44. In contrast, the phosphorylation of JAK and STAT1 increased with SOCS1 and SOCS3 expression. Therefore, it is inaccurate to describe the JAK–STAT pathway as being inhibited globally. A more plausible interpretation is that pathway remodeling is accompanied by the induction of negative feedback regulators. Likewise, increased IκBα phosphorylation together with reduced p65 phosphorylation may reflect time-dependent regulatory turnover rather than simple stabilization of IκBα. Time-resolved analyses, nuclear translocation assays, and selective pathway perturbation are required to establish the causal sequence linking CAR engagement, MerTK signaling, and suppression of inflammatory transcription.

CAR macrophages have primarily been developed for oncological applications, where engineered macrophages can combine antigen recognition, phagocytosis, and local immune modulation 17,45. The present study extends this design logic to include patients with inflammatory arthritis. Incorporation of a CD11b-targeting nanobody into the AAV2 capsid increases the transduction of bone marrow-derived macrophages from 2.74% to approximately 15%, which is consistent with the broader feasibility of nanobody-mediated AAV retargeting 21. After intra-articular administration, the CAR signal colocalized predominantly with CD11b^+^ F4/80^+^ macrophage-lineage cells and showed minimal overlap with Ly6C/G^+^ neutrophils or CD11c^+^ dendritic cells. These findings support preferential, although not absolute, targeting of macrophage-lineage cells within inflamed joints.

In collagen-induced arthritis (CIA), local anti-TNFα CAR delivery reduced paw swelling and arthritis scores, preserved periarticular bone microarchitecture, and decreased histological inflammation and osteoclast-associated damage. Joint and systemic inflammatory mediators were significantly reduced by day 14, whereas MerTK^+^ CD206^+^ macrophages increased in the treated joints. Under the specific dosing schedules tested, these effects were greater than those observed with adalimumab. This comparison should be interpreted as preclinical evidence within the present model rather than as proof of clinical superiority, because exposure, pharmacokinetics, and treatment timing did not match across modalities. Short-term histology and serum chemistry did not reveal any detectable toxicity in major organs. AAV vectors have an established and expanding clinical development landscape 46, and transgene expression can be sustained in selected tissues 47. However, neither the long-term expression in synovial macrophages nor the long-term safety was established in this study.

Several limitations define the next steps toward translation. First, local AAV-CAR was administered before booster immunization in the CIA protocol; therefore, the study modeled prevention or early intervention more closely than the treatment of established, treatment-refractory RA. Therapeutic dosing after the onset of stable arthritis, head-to-head exposure-controlled comparisons, and evaluations using additional models are required. Second, synovial fluid-conditioned experiments improve disease relevance, but do not reproduce the cellular and spatial organization of the human synovium, and samples described as originating from patients with arthritis should not be assumed to represent a uniform RA population. Donor-level heterogeneity and responses of primary human synovial macrophages should be assessed. Third, CD11b is expressed in several myeloid populations; therefore, its biodistribution, off-target transduction, and vector shedding require quantitative evaluation beyond co-localization imaging. Fourth, the durability, reversibility, and immunogenicity of repeated or sustained CAR expression remains unknown. Finally, direct loss-of-function experiments are required to separate the contributions of TNF-α depletion, lysosomal degradation, and MerTK-dependent intracellular signaling.

Within these boundaries, this study provides a mechanistically differentiated proof of concept: a pathogenic cytokine can be used not only as a target for neutralization but also as a molecular cue that activates its own cellular clearance and promotes a pro-resolving macrophage program. This coupling of cytokine removal with local immune reprogramming offers a potential route for more durable control of inflammatory arthritis and provides a modular framework for investigating other chronic inflammatory diseases driven by persistent soluble mediators.

## Conclusions

We developed a macrophage-targeted anti-TNFα CAR platform that integrates TNFR1-mediated TNF-α recognition with MerTK-driven pro-resolving signaling. Rather than merely neutralizing TNF-α, this system converts the pathogenic cytokine into a trigger for its own capture, phagocytic internalization and lysosomal degradation, while simultaneously reprogramming macrophages toward an inflammation-resolving MerTK⁺CD206^+^ phenotype. Mechanistically, anti-TNFα CAR-M activated Rac1/Cdc42/RhoA-dependent phagocytic programs and the MerTK–JAK/STAT–SOCS axis, while suppressing NF-κB, PI3K–AKT and MAPK signaling. This dual activity reduced pro-inflammatory cytokines and chemokines, increased IL-4 and IL-10, and remodeled both experimentally induced and patient synovial fluid-derived inflammatory microenvironments more effectively than TNF-α neutralization alone. Local delivery of macrophage-targeted anti-CD11b AAV2 preferentially directs CAR expression to macrophage-lineage cells in arthritic joints. In CIA mice, anti-TNFα CAR treatment attenuated local and systemic inflammation, promoted pro-resolving macrophage polarization, preserved periarticular bone microarchitecture and reduced joint pathology, with greater efficacy than adalimumab under the tested preclinical conditions and without detectable systemic toxicity. Collectively, this study establishes a conceptually distinct therapeutic strategy that couples pathogenic cytokine clearance with active immune reprogramming, providing a promising framework for the durable resolution of inflammation in rheumatoid arthritis.

## Supplementary Material

Supplementary figures.

## Data Availability

The data supporting the findings of this study are available from the corresponding author upon reasonable request.

## Abbreviations

RA: rheumatoid arthritis
CIA: collagen-induced arthritis
TNF-α: tumor necrosis factor-α
IL-1β: interleukin-1 β
IL-6: interleukin-6
IL-4: interleukin-4
IL-10: interleukin-10
PBS: Phosphate-buffered saline
ORF: open reading frame
HSPG: heparan sulfate proteoglycans
AA: amino acids
MOI: multiplicities of infection
VG: viral genomes
BMDM: bone marrow derived macrophage
CM: Conditioned Medium
SF-CM: synovial fluid-conditioned medium
ELISA: enzyme-linked immunosorbent assay
SDS-PAGE: sodium dodecyl sulfate-polyacrylamide gel electrophoresis
HRP: horseradish peroxidase
H&E: hematoxylin–eosin
IHC: Immunohistochemistry
ICC: Immunocytochemistry
NDS: normal donkey serum
RT-qPCR: reverse transcription polymerase chain Reaction
FOV: field of view
ROI: region of interest
Micro-CT: micro-computed tomography
BMD: bone mineral density
BV/TV: bone volume fraction
Tb.N: trabecular number
Tb.Sp: trabecular separation
TRAP: tartrate-resistant acid phosphatase
CQ: chloroquine
DEG: differentially expressed gene
Ada: adalimumab

## References

[1] Aletaha D, Smolen JS (2018). Diagnosis and management of rheumatoid arthritis: a review. JAMA.

[2] Gravallese EM, Firestein GS (2023). Rheumatoid arthritis - common origins, divergent mechanisms. N. Engl. J. Med.

[3] Alivernini S, MacDonald L, Elmesmari A, Finlay S, Tolusso B, Gigante MR (2020). Distinct synovial tissue macrophage subsets regulate inflammation and remission in rheumatoid arthritis. Nat. Med.

[4] Tanaka Y (2013). Next stage of RA treatment: is TNF inhibitor-free remission a possible treatment goal?. Ann. Rheum. Dis.

[5] Brennan FM, McInnes IB (2008). Evidence that cytokines play a role in rheumatoid arthritis. J. Clin. Invest.

[6] Toyoda Y, Tabata S, Kishi J, Kuramoto T, Mitsuhashi A, Saijo A (2014). Thymidine phosphorylase regulates the expression of CXCL10 in rheumatoid arthritis fibroblast-like synoviocytes. Arthritis Rheumatol.

[7] Mewar D, Wilson AG (2011). Treatment of rheumatoid arthritis with tumour necrosis factor inhibitors. Br. J. Pharmacol.

[8] Alivernini S, Peluso G, Fedele AL, Tolusso B, Gremese E, Ferraccioli G (2016). Tapering and discontinuation of TNF-α blockers without disease relapse using ultrasonography as a tool to identify patients with rheumatoid arthritis in clinical and histological remission. Arthritis Res. Ther.

[9] Alivernini S, Tolusso B, Petricca L, Bui L, Di Sante G, Peluso G (2017). Synovial features of patients with rheumatoid arthritis and psoriatic arthritis in clinical and ultrasound remission differ under anti-TNF therapy: a clue to interpret different chances of relapse after clinical remission?. Ann. Rheum. Dis.

[10] Nagy G, van Vollenhoven RF (2015). Sustained biologic-free and drug-free remission in rheumatoid arthritis, where are we now?. Arthritis Res. Ther.

[11] Guan M, Yu Q, Zhou G, Wang Y, Yu J, Yang W, Li Z (2024). Mechanisms of chondrocyte cell death in osteoarthritis: implications for disease progression and treatment. J. Orthop. Surg. Res.

[12] Gong Z, Chen L, Zhou X, Zhang C, Matičić D, Vnuk D (2025). Mxene-based photothermal-responsive injectable hydrogel microsphere modulates physicochemical microenvironment to alleviate osteoarthritis. Smart Med.

[13] Guan M, Zhang X, Li X, Liao B, Han W, Tan J (2025). Research progress of osteoarthritis treatment by low intensity pulsed ultrasound. Smart Med.

[14] DeRyckere D, Huelse JM, Earp HS, Graham DK (2023). Tam family kinases as therapeutic targets at the interface of cancer and immunity. Nat. Rev. Clin. Oncol.

[15] Kurowska-Stolarska M, Alivernini S (2022). Synovial tissue macrophages in joint homeostasis, rheumatoid arthritis and disease remission. Nat. Rev. Rheumatol.

[16] Waterborg CEJ, Beermann S, Broeren MGA, Bennink MB, Koenders MI, van Lent PLEM (2018). Protective role of the mer tyrosine kinase via efferocytosis in rheumatoid arthritis models. Front. Immunol.

[17] Klichinsky M, Ruella M, Shestova O, Lu XM, Best A, Zeeman M (2020). Human chimeric antigen receptor macrophages for cancer immunotherapy. Nat. Biotechnol.

[18] Brand DD, Latham KA, Rosloniec EF (2007). Collagen-induced arthritis. Nat. Protoc.

[19] Yu D, Ye X, Che R, Wu Q, Qi J, Song L (2017). Fgf21 exerts comparable pharmacological efficacy with adalimumab in ameliorating collagen-induced rheumatoid arthritis by regulating systematic inflammatory response. Biomed. Pharmacother.

[20] Ying W, Cheruku PS, Bazer FW, Safe SH, Zhou B (2013). Investigation of macrophage polarization using bone marrow derived macrophages. J. Vis. Exp.

[21] Eichhoff AM, Börner K, Albrecht B, Schäfer W, Baum N, Haag F (2019). Nanobody-enhanced targeting of AAV gene therapy vectors. Mol. Ther. Methods Clin. Dev.

[22] Wajant H, Siegmund D (2019). TNFR1 and TNFR2 in the control of the life and death balance of macrophages. Front. Cell Dev. Biol.

[23] D'Alessio A, Al-Lamki RS, Bradley JR, Pober JS (2005). Caveolae participate in tumor necrosis factor receptor 1 signaling and internalization in a human endothelial cell line. Am. J. Pathol.

[24] Boulter E, Garcia-Mata R, Guilluy C, Dubash A, Rossi G, Brennwald PJ, Burridge K (2010). Regulation of rho GTPase crosstalk, degradation and activity by rhogdi1. Nat. Cell Biol.

[25] Hayer S, Vervoordeldonk MJ, Denis MC, Armaka M, Hoffmann M, Bäcklund J (2021). 'Smash' recommendations for standardised microscopic arthritis scoring of histological sections from inflammatory arthritis animal models. Ann. Rheum. Dis.

[26] Mao Y, Finnemann SC (2015). Regulation of phagocytosis by rho GTPases. Small GTPases.

[27] Jia N, Gao Y, Li M, Liang Y, Li Y, Lin Y (2023). Metabolic reprogramming of proinflammatory macrophages by target delivered roburic acid effectively ameliorates rheumatoid arthritis symptoms. Signal Transduct. Target. Ther.

[28] Zizzo G, Hilliard BA, Monestier M, Cohen PL (2012). Efficient clearance of early apoptotic cells by human macrophages requires M2c polarization and MerTK induction. J. Immunol.

[29] Zhao XN, Li YN, Wang YT (2016). Interleukin-4 regulates macrophage polarization via the MAPK signaling pathway to protect against atherosclerosis. Genet. Mol. Res.

[30] Wang Q, Jiang H, Li Y, Chen W, Li H, Peng K (2017). Targeting NF-kB signaling with polymeric hybrid micelles that co-deliver siRNA and dexamethasone for arthritis therapy. Biomaterials.

[31] Liu C, He L, Wang J, Wang Q, Sun C, Li Y (2020). Anti-angiogenic effect of Shikonin in rheumatoid arthritis by downregulating PI3K/AKT and MAPKs signaling pathways. J. Ethnopharmacol.

[32] Weber BN, Giles JT, Liao KP (2023). Shared inflammatory pathways of rheumatoid arthritis and atherosclerotic cardiovascular disease. Nat. Rev. Rheumatol.

[33] Huelse JM, Fridlyand DM, Earp S, DeRyckere D, Graham DK (2020). MerTK in cancer therapy: Targeting the receptor tyrosine kinase in tumor cells and the immune system. Pharmacol. Ther.

[34] Smolen JS, Aletaha D, McInnes IB (2016). Rheumatoid arthritis. Lancet.

[35] Tascilar K, Hagen M, Kleyer A, Simon D, Reiser M, Hueber AJ (2021). Treatment tapering and stopping in patients with rheumatoid arthritis in stable remission (retro): a multicentre, randomised, controlled, open-label, phase 3 trial. Lancet Rheumatol.

[36] Emery P, Burmester GR, Naredo E, Sinigaglia L, Lagunes I, Koenigsbauer F, Conaghan PG (2020). Adalimumab dose tapering in patients with rheumatoid arthritis who are in long-standing clinical remission: results of the phase IV PREDICTRA study. Ann. Rheum. Dis.

[37] Tanna CE, Goss LB, Ludwig CG, Chen PW (2019). Arf GAPs as regulators of the actin cytoskeleton-an update. Int. J. Mol. Sci.

[38] Cai B, Kasikara C, Doran AC, Ramakrishnan R, Birge RB, Tabas I (2018). MerTK signaling in macrophages promotes the synthesis of inflammation resolution mediators by suppressing CaMKII activity. Sci. Signal.

[39] Sayegh S, El Atat O, Diallo K, Rauwel B, Degboé Y, Cavaignac E (2019). Rheumatoid synovial fluids regulate the immunomodulatory potential of adipose-derived mesenchymal stem cells through a TNF/NF-κB-dependent mechanism. Front. Immunol.

[40] Degboé Y, Rauwel B, Baron M, Boyer J-F, Ruyssen-Witrand A, Constantin A, Davignon J-L (2019). Polarization of rheumatoid macrophages by TNF targeting through an IL-10/STAT3 mechanism. Front. Immunol.

[41] Opal SM, DePalo VA (2000). Anti-inflammatory cytokines. Chest.

[42] Murray PJ, Allen JE, Biswas SK, Fisher EA, Gilroy DW, Goerdt S (2014). Macrophage activation and polarization: nomenclature and experimental guidelines. Immunity.

[43] Ding Q, Hu W, Wang R, Yang Q, Zhu M, Li M (2023). Signaling pathways in rheumatoid arthritis: implications for targeted therapy. Signal Transduct. Target. Ther.

[44] Moelants EAV, Mortier A, Van Damme J, Proost P (2013). Regulation of TNF-α with a focus on rheumatoid arthritis. Immunol. Cell Biol.

[45] Lei A, Yu H, Lu S, Lu H, Ding X, Tan T (2024). A second-generation M1-polarized CAR macrophage with antitumor efficacy. Nat. Immunol.

[46] Kuzmin DA, Shutova MV, Johnston NR, Smith OP, Fedorin VV, Kukushkin YS (2021). The clinical landscape for AAV gene therapies. Nat. Rev. Drug Discov.

[47] Muhuri M, Levy DI, Schulz M, McCarty D, Gao G (2022). Durability of transgene expression after rAAV gene therapy. Mol. Ther.

