# Supplementary material for: Targeted phagocytosis of TNF-α by CAR macrophages restores immune homeostasis in arthritis
Source: Theranostics. 2026 Aug 24;16(15):9007–29. doi: 10.7150/thno.132172 (PMC13549430; doi:10.7150/thno.132172)
Supplement: Supplementary file 1 [file thnov16p9007s1.pdf]

A

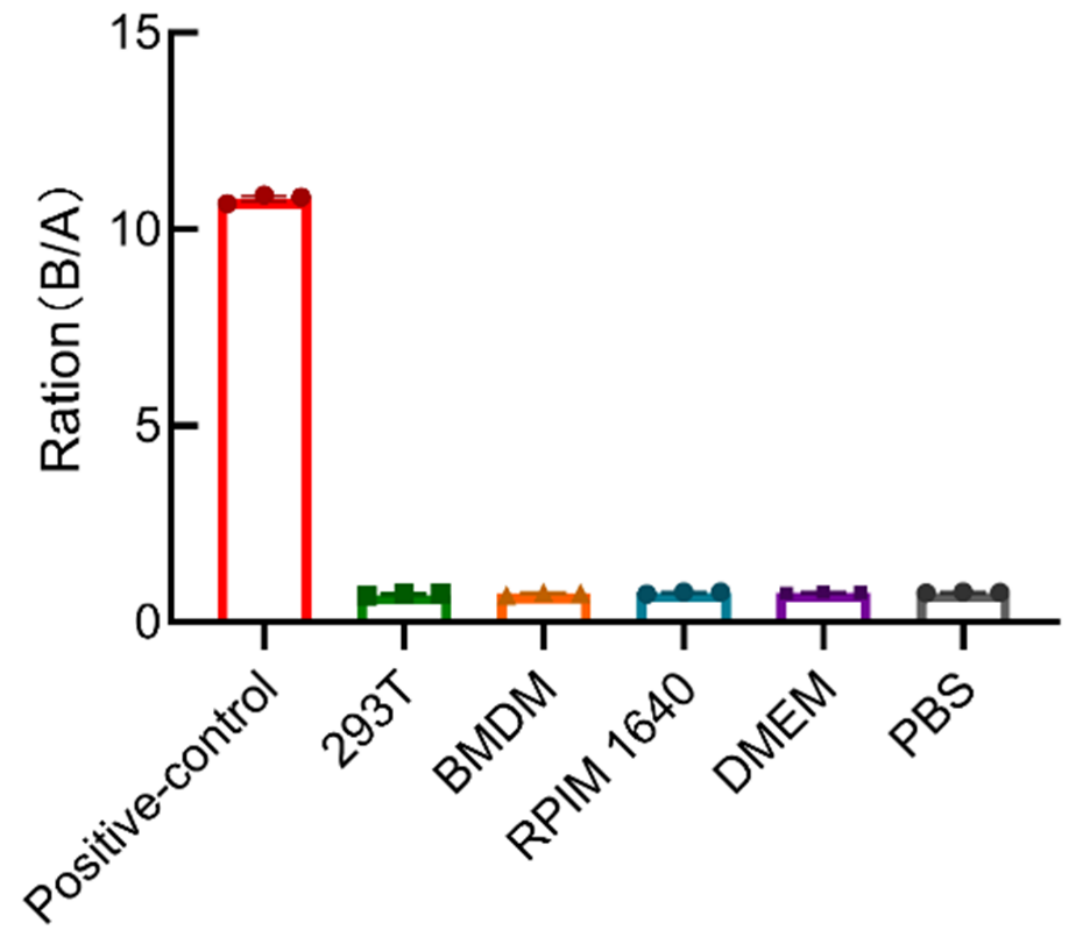

B

Sample	Positive-control	HEK-293T	BMDM	RPIM 1640	DMEM	PBS
$A_{average}$	111043	23717.33	36605.33	25829.3333	27112	23040.33
$B_{average}$	1192329	16778	26726.67	19445.6667	20482.33	16450.33
$Ratio_{average}$ (B/A)	10.77819	0.707406	0.730898	0.75486884	0.755405	0.71297

Figure S1. Detection of Mycoplasma Contamination Using the Myco-Lumi™ Luminescent Assay

(A) The resulting B/A ratios (right) in HEK293T cells, BMDMs, and common reagents (RPMI 1640, DMEM, PBS). **(B)** Data represents the average of measurements from three independent samples per group. The Myco-Lumi™ Luminescent Mycoplasma Detection Kit utilizes mycoplasma-specific enzymatic activity to detect contamination through an ATP-dependent luminescent reaction catalyzed by luciferase. The assay determines mycoplasma presence by comparing changes in ATP levels before and after reagent addition. The entire procedure consists of two simple steps and takes approximately 15 minutes. In Step 1, Reagent A is added to the sample, and the initial luminescence (Value A) is measured after 5 minutes, representing the background ATP level. In Step 2, Reagent B is introduced, and luminescence (Value B) is

measured after 10 minutes. If mycoplasma is present, its specific enzymes convert ADP in Reagent B to ATP, increasing the total ATP detected. Contamination is determined by calculating the ratio of Value B to Value A: a ratio > 1.2 indicates mycoplasma contamination (with higher ratios reflecting greater contamination), while a ratio < 0.9 confirms its absence. n = 3. Data are presented as mean ± S.E.M and statistical analysis was performed using one-way ANOVA.

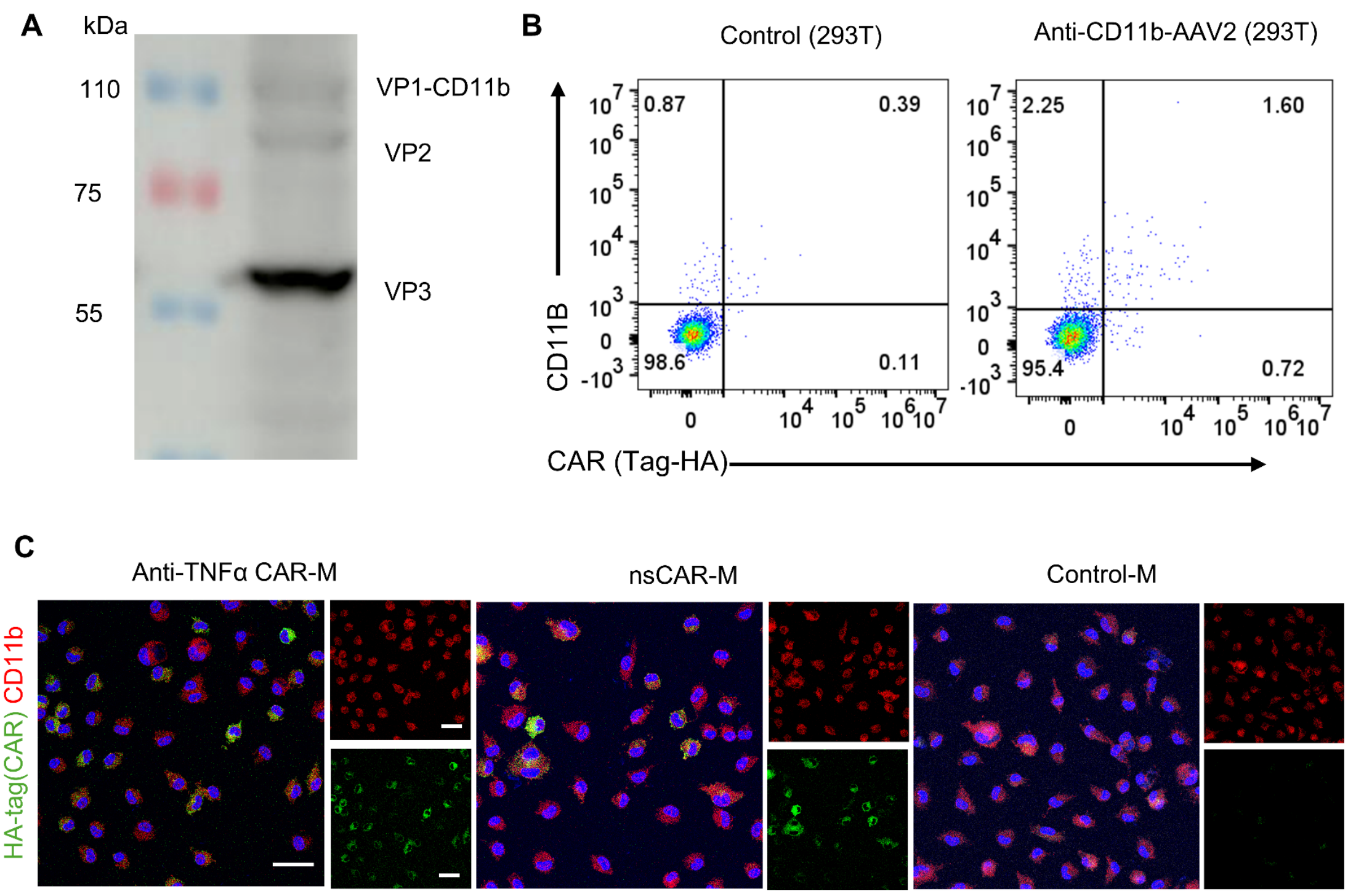

Figure S2. anti-CD11b AAV improve the transduction efficiency of anti-TNFα CAR to BMDM.

(A) Anti-CD11b-AAV capsid proteins were analyzed by Western blotting using an antibody recognizing a shared C-terminal epitope in all capsid proteins. **(B)** Transduction efficiency of anti-TNFα CAR delivered by anti-CD11b-AAV in 293T cells was evaluated by flow cytometry. **(C)** Representative confocal images showing anti-TNFα CAR and nsCAR protein

expression (HA, green) in BMDMs (CD11b, red) after anti-CD11b-AAV infection. Scale bar, 25 μm.

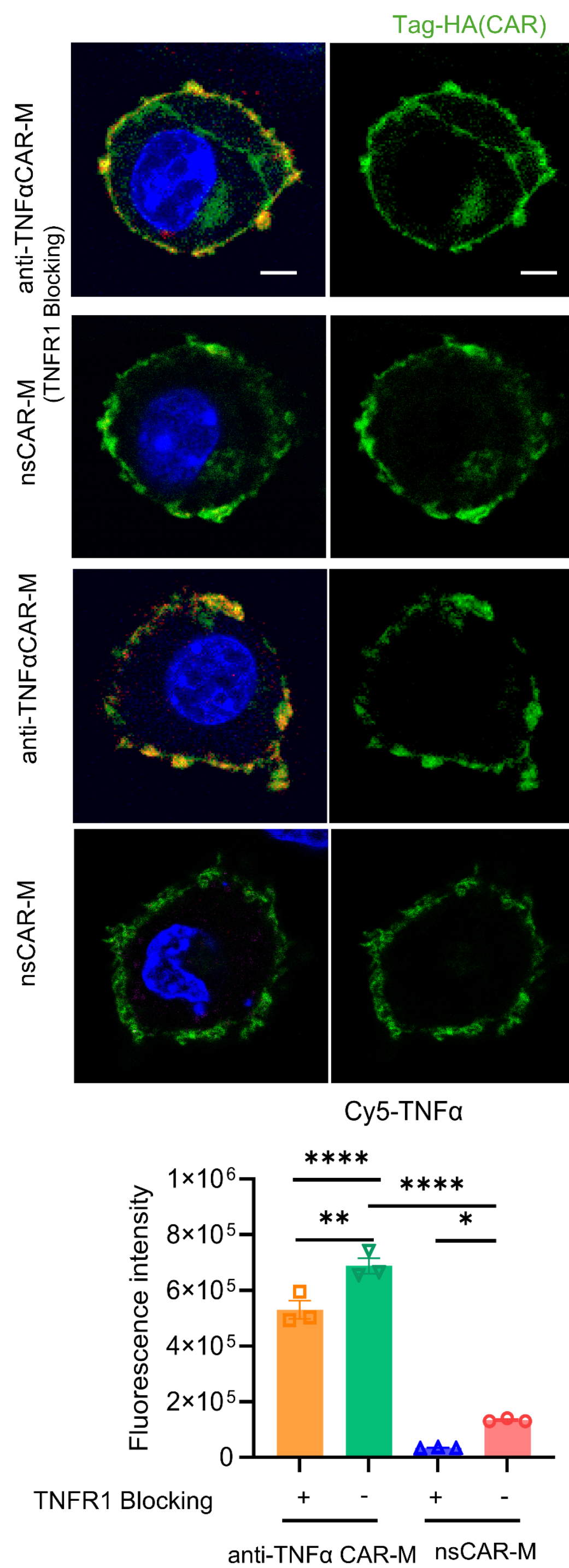

Figure S3. TNFR1 blockade confirms CAR-dependent TNF-α binding and uptake by CAR-M.

BMDMs were pre-treated with an anti-TNFR1 blocking antibody to inhibit endogenous TNFR1-mediated TNF-α uptake before transfection of CAR or nsCAR. Cells were subsequently exposed to Cy5-labeled TNF-α, and Cy5 fluorescence was analyzed. Following TNFR1 blockade, anti-TNFα CAR-M still showed a much stronger Cy5-TNF-α signal than nsCAR-M, whereas nsCAR-M displayed only low basal fluorescence. Fluorescence exposure settings were optimized to reduce background signal in nsCAR-M controls. Scale bar represents 5 μm. n = 4. Data are presented as mean ± S.E.M and statistical analysis was performed using one-way ANOVA.

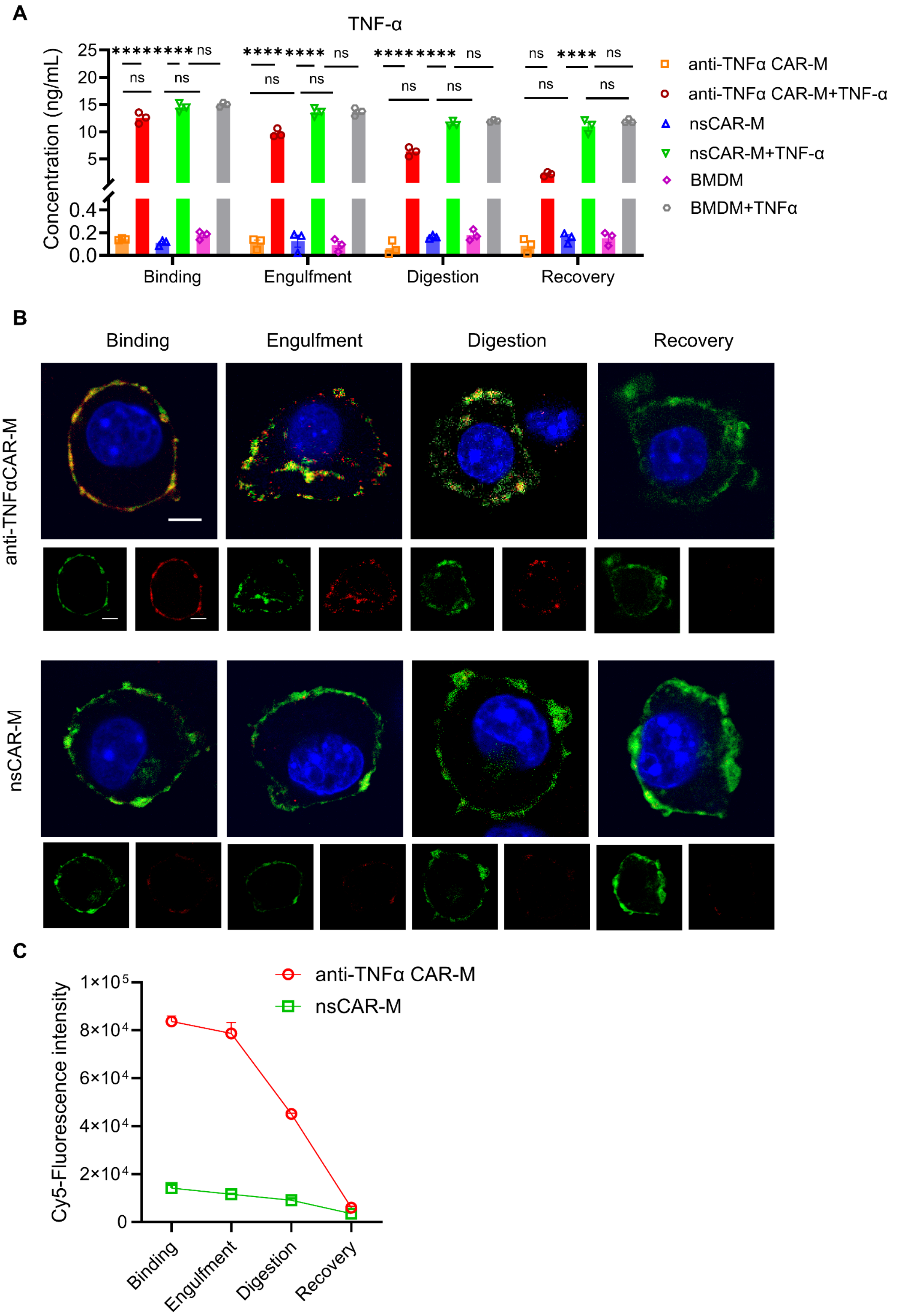

Figure S4. Endogenous TNFα neutralization and Cy5-TNFα uptake kinetics in CAR-M and nsCAR-M.

(A) TNFα levels in BMDM, nsCAR-M and CAR-M culture supernatants were measured by

ELISA before and after addition of exogenous Cy5-TNFα. Data are presented as mean ± S.E.M. **(B)** Representative ICC images of Cy5-TNFα uptake and degradation in CAR-M and nsCAR-M after pretreatment with a TNFα-neutralizing antibody. Cells were washed to remove unbound antibody before incubation with exogenous Cy5-TNFα (CAR, green; cy5-TNFα, red). Scale bars represent 5 μm. **(C)** Quantification of Cy5 fluorescence intensity showing the degradation kinetics of exogenous Cy5-TNFα in CAR-M and nsCAR-M under endogenous TNFα-neutralized conditions. n = 3. Data are presented as mean ± S.E.M and statistical analysis was performed using one-way ANOVA.

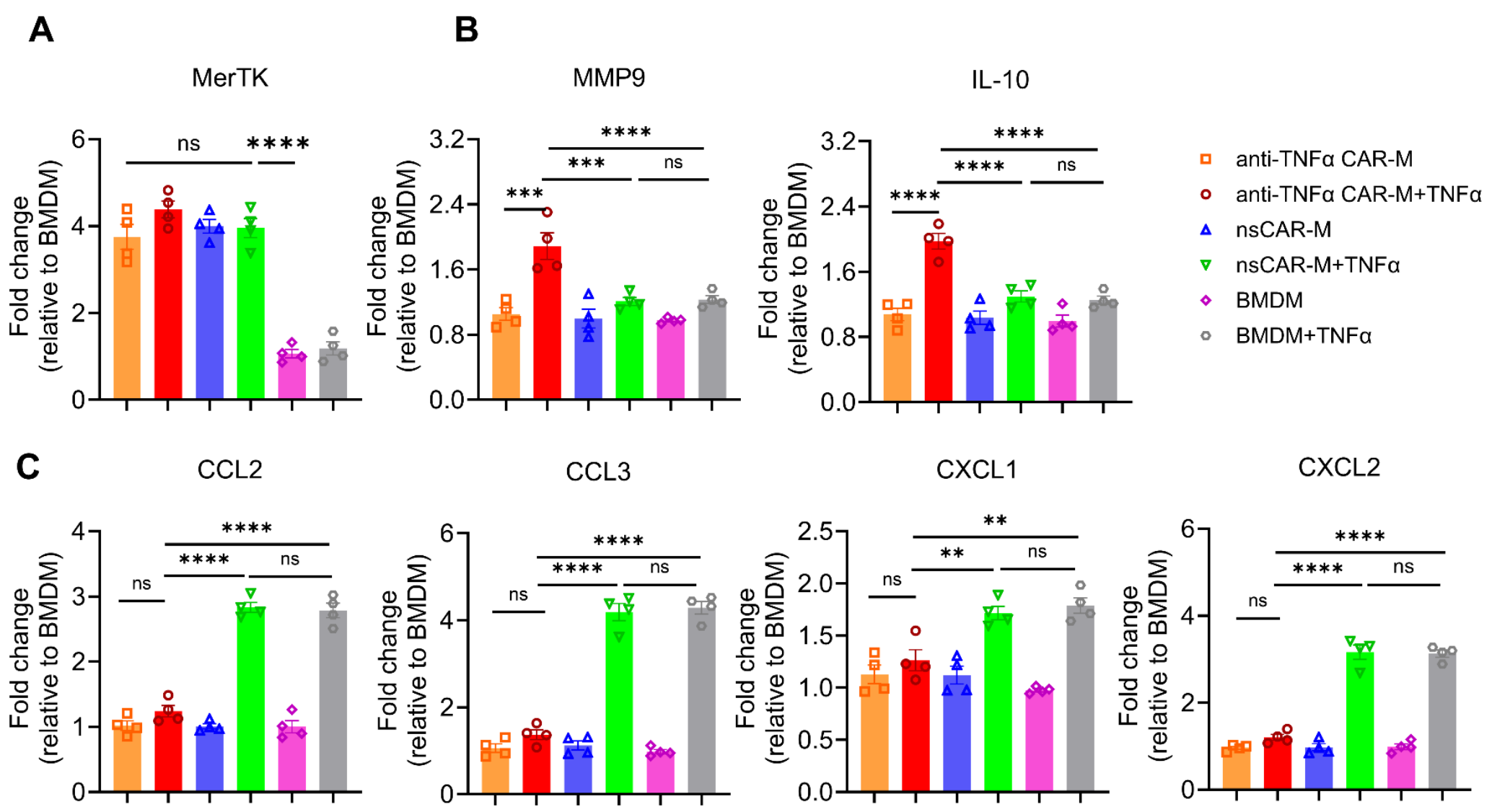

Figure S5. qPCR validation of selected RNA-seq-derived DEGs in BMDMs, nsCAR-M, and CAR-M after TNF-α stimulation.

(A) qPCR measurement of MerTK expression in nsCAR-M and CAR-M. **(B)** qPCR analysis of M2-like or tissue-remodeling genes, including IL-10 and MMP9. **(C)** qPCR analysis of inflammatory chemokine genes, including CCL2, CCL3, CXCL1, and CXCL2. Gene expression was normalized to β-actin and expressed relative to the BMDM control group. n =

3. Data are shown as mean ± S.E.M and statistical analysis was performed using one-way ANOVA.

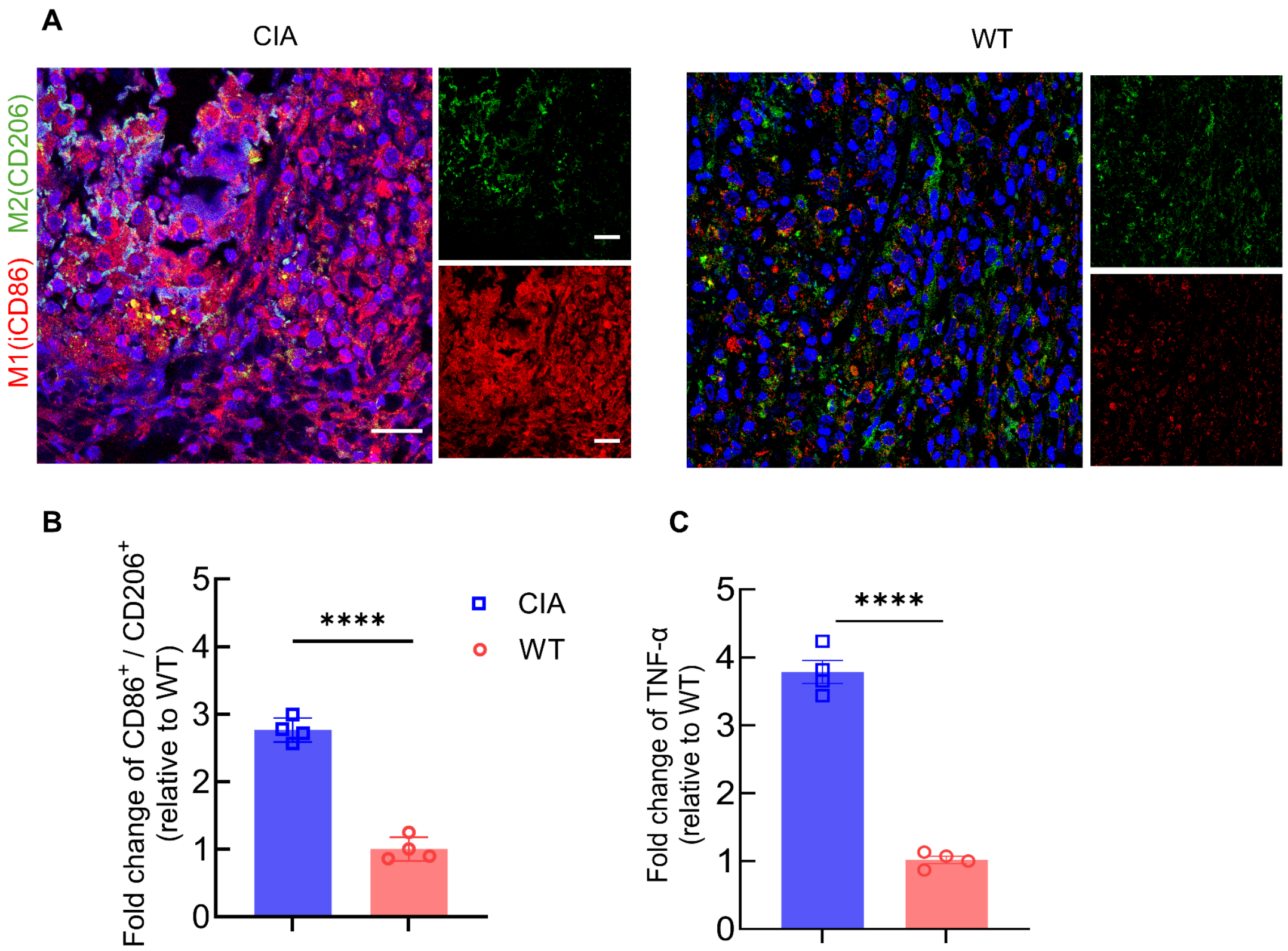

Figure S6. The increased number of M1 macrophages and the mRNA level of TNF-α in the ankle joint region of CIA mice.

(A) Representative confocal images showing M1-like macrophages (CD86, red) and M2-like macrophages (CD206, blue) in ankle joints from CIA and WT mice. Scale bar, 25 µm. **(B)** Quantification of fluorescence intensity from **(A)** using ImageJ. n = 4. Data are presented as mean ± S.E.M and statistical analysis was performed using one-way ANOVA. **(C)** TNF-α expression in ankle joints from CIA and WT mice was measured by qPCR. n = 4. Data are presented as mean ± S.E.M and statistical analysis was performed using one-way ANOVA.

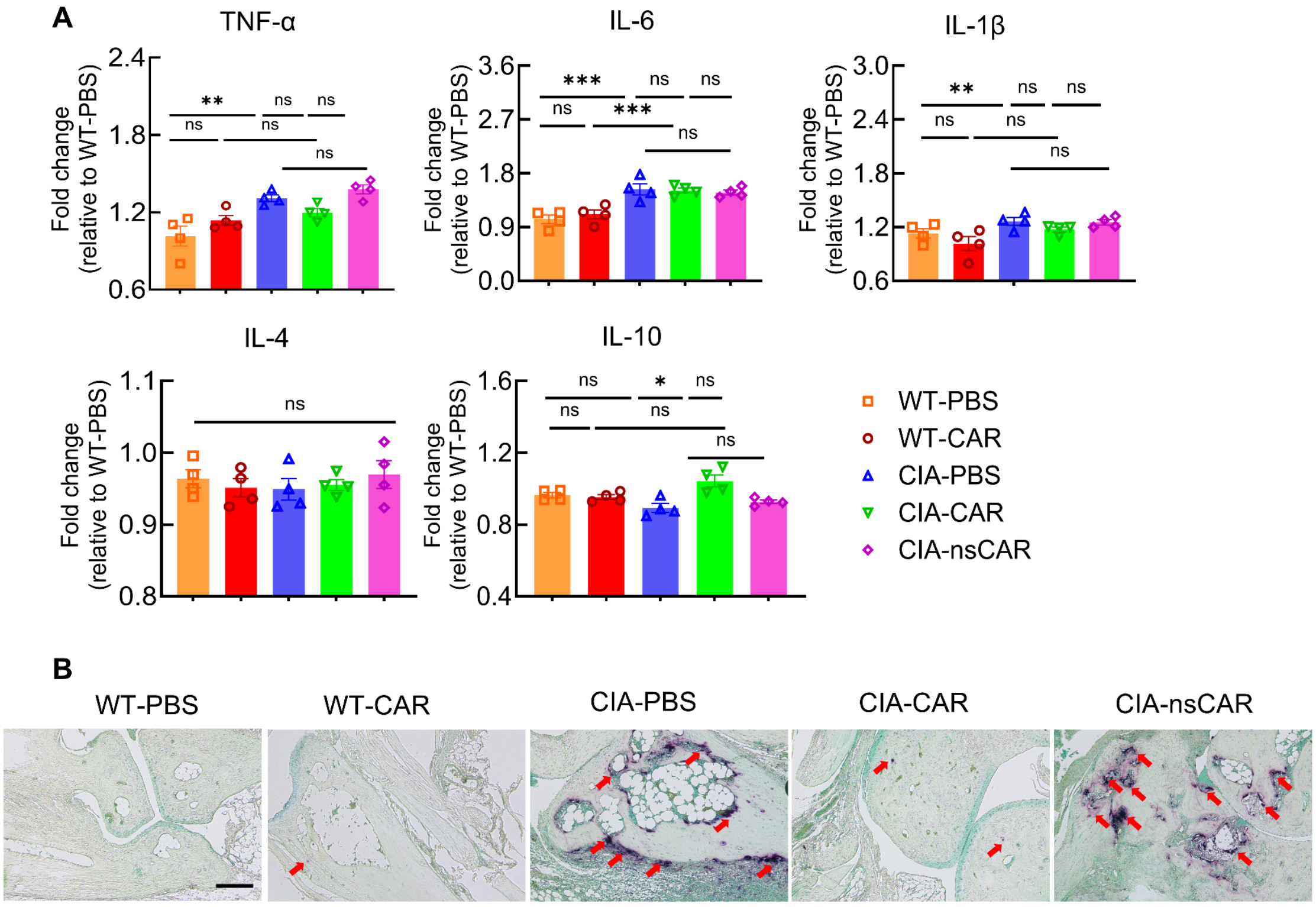

Figure S7. Inflammatory cytokine profiles and TRAP-positive osteoclast accumulation in CIA mice

(A) Serum levels of TNF-α, IL-6, IL-1β, IL-4, and IL-10 were measured by ELISA in five mouse groups on day 7. n = 4. Data are presented as mean ± S.E.M relative to WT-PBS, and statistical analysis was performed using one-way ANOVA. **(B)** Representative TRAP-stained ankle joint sections from CIA-PBS and nsCAR-treated mice. Increased numbers of TRAP-positive osteoclasts, together with narrowing of the ankle joint cavity, indicate inflammation-related bone erosion and joint destruction. Red arrows mark TRAP-positive osteoclasts. Scale bar, 200 µm.

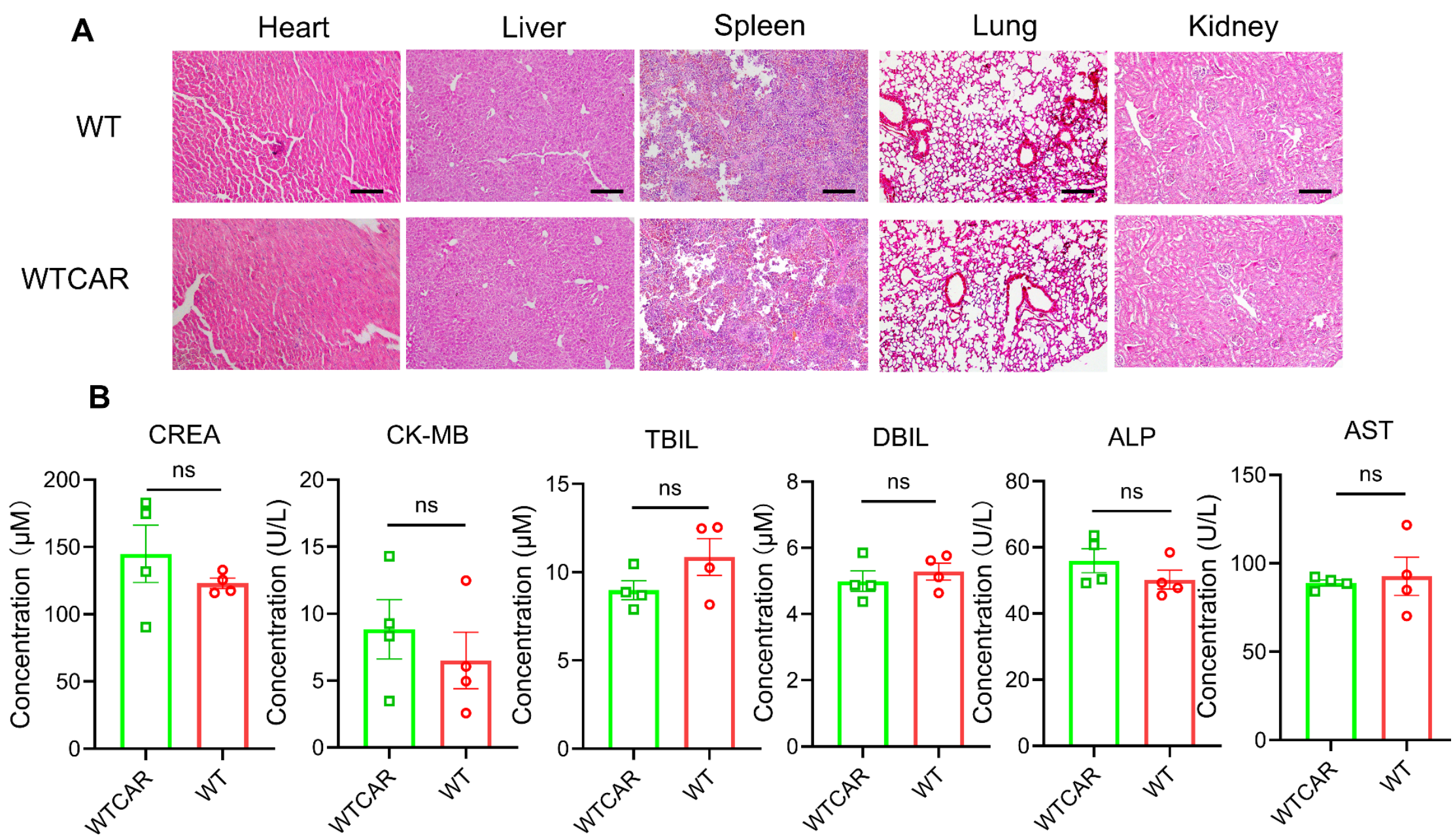

Figure S8. Biosafety evaluation of local AAV-mediated anti-TNFα CAR delivery.

(A) Representative H&E-stained sections of major organs, including the heart, liver, spleen, lung, and kidney, from WT-PBS and WT-CAR mice. Scale bar, 200 μm. **(B)** Serum biochemical assessment of CREA, CK-MB, TBIL, DBIL, ALP, and AST in WT-PBS and WT-CAR mice. n = 4. Data are presented as mean ± S.E.M and statistical analysis was performed using one-way ANOVA.

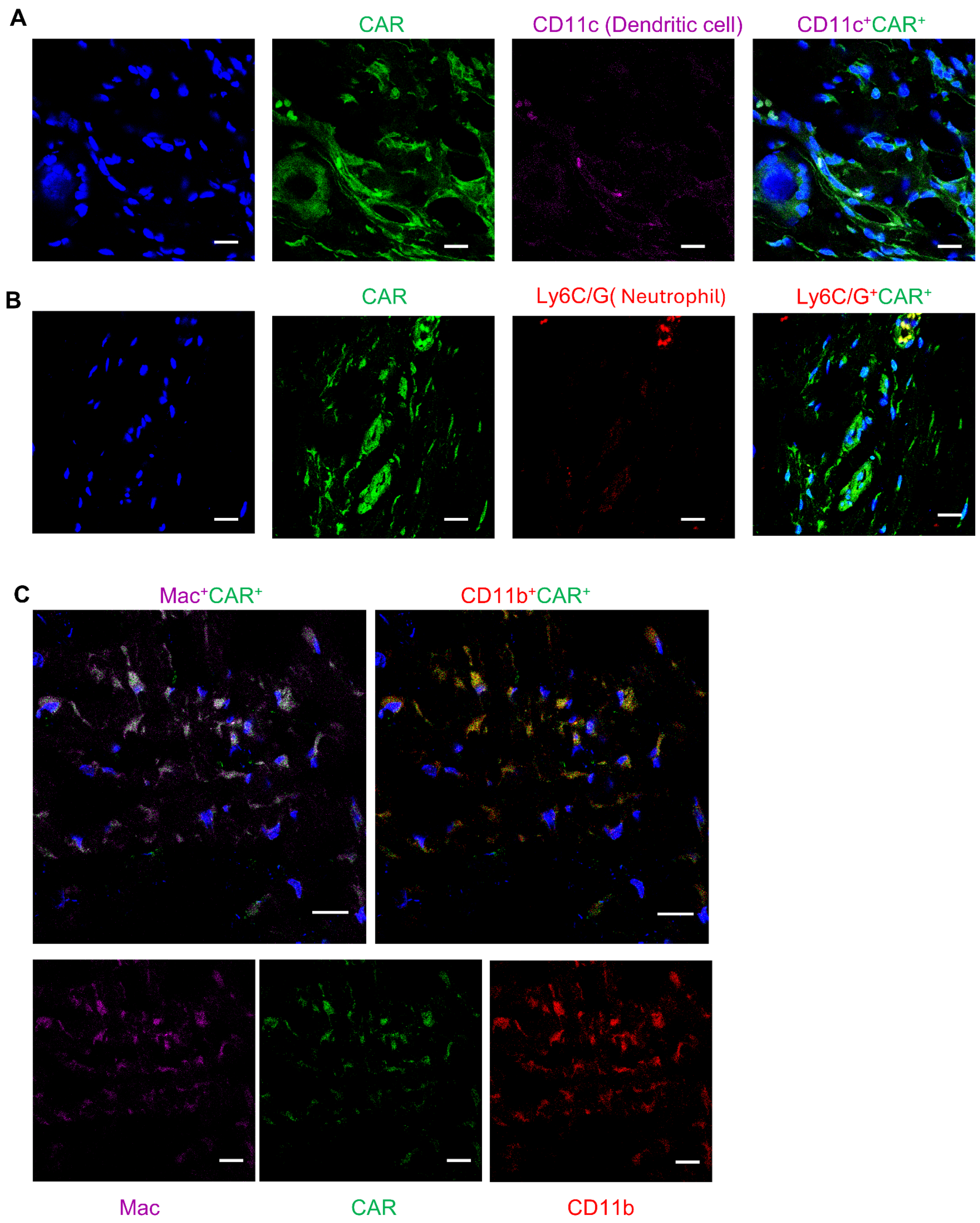

Figure S9. Preferential enrichment of anti-CD11b AAV-delivered CAR in macrophage-lineage cells within arthritic joints.

(A-C) Representative immunofluorescence images of arthritic joint sections showing CAR staining (green) in relation to **(A)** Ly6C/G^{+} neutrophils, **(B)** CD11c^{+} dendritic cells, and **(C)** CD11b^{+}F4/80^{+} macrophage-lineage cells. CAR fluorescence displayed minimal colocalization

with neutrophils and dendritic cells, whereas it was predominantly associated with $CD11b^+$ $F4/80^+$ macrophage-lineage cells. Scale bar, 25 μm.
